# Chromatin compartment shifting is recurrent and associated with epigenetic reprogramming in prostate cancer

**DOI:** 10.1016/j.isci.2026.118155

**Published:** 2026-11-06

**Authors:** Tuomo Virtanen, Ebrahim Afyounian, Riikka Nurminen, Joonas Uusi-Mäkelä, Antti Kiviaho, Joonatan Sipola, Francesco Tabaro, Juha Kesseli, Teuvo Tammela, Teemu J. Murtola, Alfonso Urbanucci, Tapio Visakorpi, Kirsi Rautajoki, Matti Nykter

**Affiliations:** 1 Center for Cancer Eradication Research, Faculty of Medicine and Health Technology, Tampere University and TAYS Cancer Center, Tampere, Finland; 2 Department of Urology, TAYS Cancer Centre, Tampere, Finland; 3 Finnish Cancer Institute, Helsinki, Finland; 4 Department of Tumor Biology, Institute for Cancer Research, Oslo University Hospital, Oslo, Norway; 5 Fimlab Laboratories Ltd, Tampere, Finland; 6 Data Science Center, Epigenetics and Neurobiology Unit, EMBL Rome, Monterotondo, Italy

**Keywords:** prostate cancer, Hi-C, chromatin conformation, AR

## Abstract

Three-dimensional chromatin organization shapes gene regulation by partitioning the genome into transcriptionally active and inactive compartments, with implications for prostate carcinogenesis. We integrated Hi-C, ATAC-seq, and transcriptomic data from 18 clinical samples representing benign prostatic hyperplasia, primary prostate cancer (PC), and castration-resistant prostate cancer (CRPC). Using a computational framework accounting for copy number variation and tumor purity, we characterized A/B chromatin compartment organization in aneuploid tumors. We identified recurrent shifts from inactive B to active A compartments in both PC and CRPC, indicating shared chromatin reorganization across disease stages. These regions were enriched for prostate cancer-associated gene expression, including the biomarker *PCA3*, active epigenetic marks, and accessible chromatin bound by prostate lineage transcription factors, including AR, FOXA1, and HOXB13. These findings link higher-order chromatin organization to transcriptional and epigenetic rewiring, highlighting compartment shifts as a recurring feature of prostate carcinogenesis.

## Highlights

• Tumor-tissue Hi-C analysis accounting for tumor purity and copy number variation
• Recurrent chromatin compartment shifts occur in prostate cancer
• Compartment shifts are associated with prostate cancer specific gene activation
• Active compartments are enriched for prostate lineage transcription factors

## Graphical abstract

**Figure:**
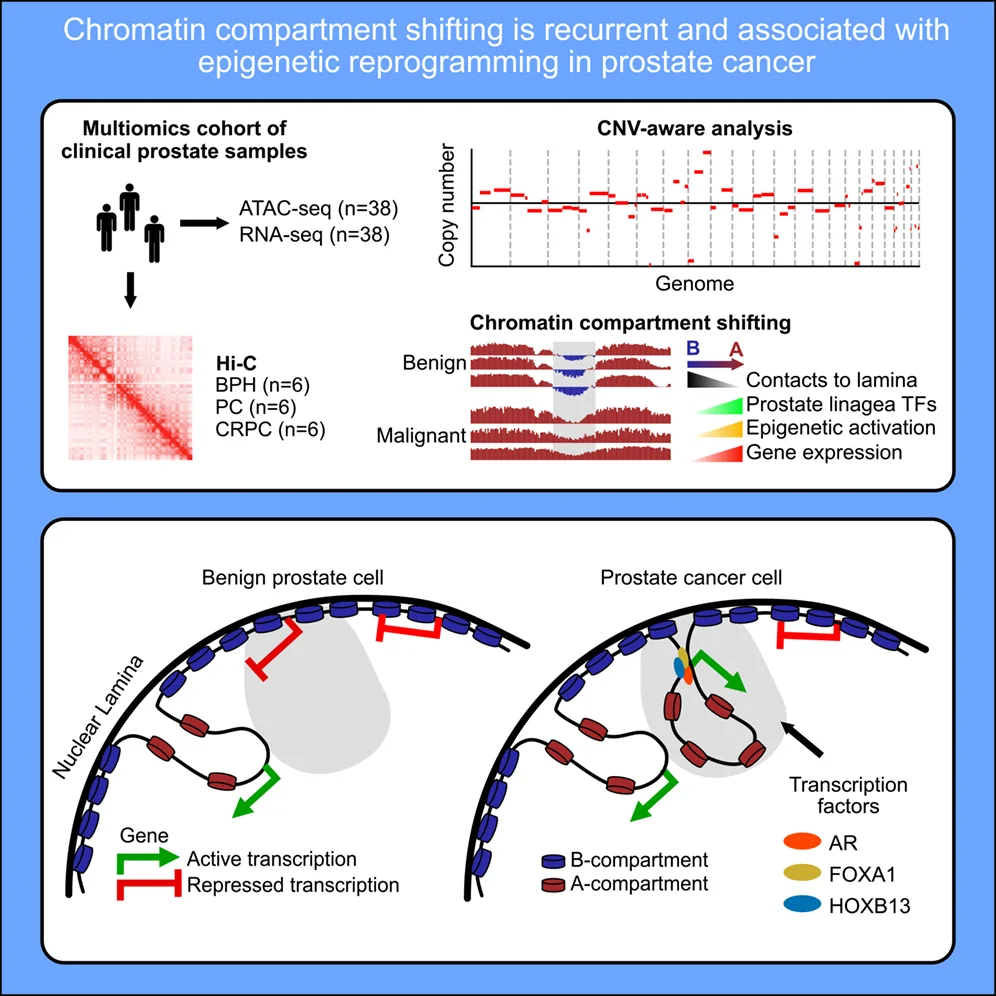

## Teaser

Genomics; Epigenetics; Systems biology; Cancer

## Introduction

Advances in genomic studies have shown that primary prostate cancer exhibits a relatively low mutational burden, with only a limited number of recurrent driver alterations identified to date, yet the somatic events initiating tumorigenesis remain incompletely understood.^1^ Growing evidence points to epigenetic dysregulation as a central driver in prostate cancer tumorigenesis and resistance to primary therapies.^2^ A better understanding of these mechanisms is essential for improving patient care, from prognostic assessment to therapeutic intervention. Different layers of the epigenome—such as DNA methylation, histone modifications, and transcription factor (TF) binding —have been extensively characterized in prostate cancer.^3^^,^^4^^,^^5^^,^^6^^,^^7^ However, the three-dimensional (3D) conformation of chromatin remains comparatively understudied.^8^^,^^9^^,^^10^^,^^11^^,^^12^^,^^13^ This chromatin conformation governs enhancer-promoter communication and shapes large-scale compartmental folding between the nuclear lamina and nuclear interior. Advances in sequencing methods such as high-throughput chromatin conformation capture sequencing (Hi-C) has enabled genome-wide mapping of physical interactions between genomic regions, revealing the spatial organization of the genome.^14^ Hi-C sequencing studies across human cell types and tissues have revealed that the genome is spatially organized into two major compartments: active (A) and inactive (B). Functionally, B compartments are enriched for heterochromatin, show repressed gene expression, and are often associated with the nuclear lamina, while A compartments correspond to transcriptionally active euchromatin that tends to occupy the nuclear interior.^14^^,^^15^

Chromatin compartments can shift under normal biological processes such as development and cell differentiation, partitioning the genome into transcriptionally active and inactive domains, thereby potentially influencing cellular identity and helping maintain lineage-specific transcriptional programs by selectively activating or silencing genomic regions.^16^ Profiling of compartmentalization across 21 human tissues and cell lines demonstrated that approximately 60% of the genome exhibits compartment variability between cell types, underscoring the dynamic nature of these structures.^17^ Given that tumorigenesis involves extensive remodeling of cellular identity and transcription, it is plausible that compartment shifting contributes to the dysregulation of gene expression programs in cancer. Consistent with this, multiple studies have reported widespread compartment shifting across diverse cancer types.^18^^,^^19^^,^^20^

To study how chromatin compartments are altered across disease states, we generated a Hi-C dataset from clinical prostate tissue samples spanning benign prostatic hyperplasia (BPH), primary untreated prostate cancer (PC), and locally recurrent castration-resistant prostate cancer (CRPC). We utilized a computational pipeline that rigorously controls tumor purity, copy number alterations, and structural variation, enabling more accurate analysis of 3D chromatin conformation within malignant cells. Using these resources, we investigated how 3D chromatin changes in different states of the disease. We highlight A/B compartment structure alterations and demonstrate the association of compartmental changes to the transcriptional dysregulation in prostate cancer.

## Results

### Nuclear lamina association shapes chromatin compartmentalization

To study the chromatin conformation alterations in prostate cancer, we performed Hi-C sequencing on 18 clinical prostate samples from 18 patients with BPH (*n* = 6), PC (*n* = 6) or CRPC (*n* = 6). These tumors are part of previously published epigenetic datasets of 39 samples consisting of RNA sequencing (*n* = 38) and chromatin accessibility (assay for transposase-accessible chromatin using sequencing, ATAC-seq) (*n* = 38), providing complementary data from matched samples^21^^,^^22^ (Table S1). After sequencing and bioinformatics pipeline, we reached an average of 836 M read pairs per sample (range, 324 M–1,581 M) (Table S2). Replicate samples were highly concordant (Table S3). Tumor purity and copy number variation pose major challenges in cancer tissue analysis, as both can distort epigenomic measurements. Variability in cellular composition and DNA dosage can obscure true regulatory signals, complicating the accurate interpretation of epigenomic signals in cancer. To tackle these confounding issues, we established a pipeline to normalize their effects in Hi-C data (see STAR Methods). We estimated the tumor purities from copy number profiles acquired directly from Hi-C data. The tumor purity varied between 16% and 80% (mean: 45%, median: 41%, Figure S1; Table S1).

After correcting for biases introduced by copy number variation (Figure S2), A/B compartments were identified using the first principal component (hereafter referred to as AB-score) of the Pearson correlation matrix at 100 kb resolution (Figure 1A; Table S4). AB-scores were highly correlated between samples (Figure 1B; Figure S3). The average AB-score across BPH samples was used to define the reference A/B compartmentalization in prostate cells. Based on this reference, the genome was partitioned into 1,269 Mb of A compartments and 1,386 Mb of B compartments. Sex chromosomes and blacklisted genomic regions (Table S5) were excluded from the analysis. As previously reported, B compartments have lower density of genes, GC content, and gene expression as well as higher density of mutations (Figure S4).^10^^,^^14^ We used publicly available lamin-B1 ChIP-Seq data to validate the association of B compartments with the nuclear lamina.^24^ Consensus lamin-binding sites were generated by identifying regions consistently enriched for lamin-B1 across multiple cell types. The average lamin-B1 ChIP-seq signal intensity was significantly higher in B compartments than in A compartments (Figure S4) and the annotated lamin-binding sites were consistent with B compartment identity. Notably, 67% of the 100 kb bins in B compartments overlapped with consensus lamin-binding sites, compared to only 1% in A compartments (Figure 1B). For each bin, we then calculated the average Hi-C contacts with these lamin anchors and observed a strong correlation between AB-scores and lamin association (Figure 1C). Together, these results demonstrate that the AB-score serves as a reliable proxy for lamin association, with B compartments localized at the nuclear lamina.

**Figure 1:**
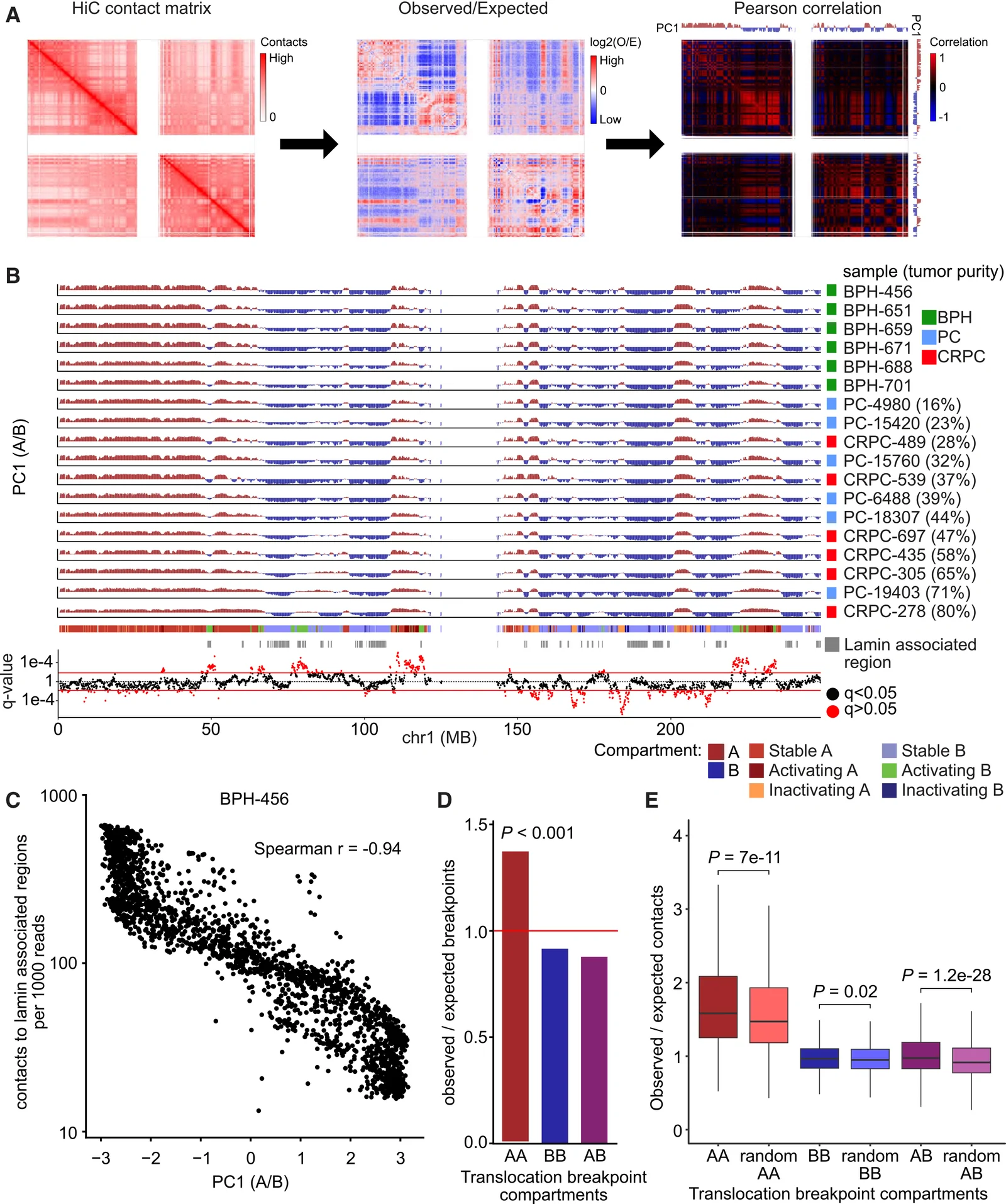
A/B compartments across the prostate tissue cohort (A) Illustration of the detection of A/B compartments. The contact matrix is normalized by the expected number of contacts (O/E = observed/expected) at each genomic distance. Custom copy number variation bias correction is applied as described in STAR Methods (Figure S2) and Pearson correlation of the normalized contacts is calculated between all 100 kb bins in the chromosome. Finally, A/B score is calculated by taking the first principal component (PC1) of the Pearson matrix. (B) Overview of the A/B scores in chromosome 1 across all samples. Prostate cancer (PC) and castration resistant prostate cancer (CRPC) samples are ordered by their tumor fraction in ascending order from top to bottom. Consensus lamin binding sites are shown in gray. The bottom track shows the –log10 of adjusted *p* values from differential compartment analysis and the color annotations refer to the observed compartment shifts switches (as detailed in next section). Red lines indicate the significance threshold corresponding to an adjusted *p* value of 0.05. BPH, benign prostatic hyperplasia. (C) Association between the A/B score and contact frequency to regions associated with the nuclear lamina. Each point represents a 100 kb bin in chromosome 1. (D) Interchromosomal translocations preferentially occur between A compartments. Bar plot showing the ratio of observed to expected frequencies for translocation-linked compartment pairs. A total of 15,526 interchromosomal translocations from 101 samples are derived from a public dataset of castration resistant prostate cancer samples (CRPC).^23^ “AA” indicates both breakpoints originate in A compartments, “BB” indicates both originate in B compartments, and “AB” indicates breakpoints arise from different compartments. Expected frequencies and empirical *p* values were obtained by randomly shuffling compartment labels 1000 times within each chromosome. (E) Translocations between A compartments show stronger interactions than those between B compartments or mixed (A and B) compartments. For each translocation, the normalized interaction frequency was calculated from interchromosomal contact matrices at 1 Mb resolution averaged across the BPH samples. A random background was generated by pairing observed translocation breakpoints randomly to assess expected contact frequencies between different compartments. Boxplots show the median as the central line, with whiskers extending to 1.5 times the interquartile range from the lower and upper quartiles. *p* values are calculated using the Wilcoxon signed-rank test.

### Interchromosomal translocations are guided by chromatin conformation

Structural variants have been shown to be influenced by spatial proximity.^10^^,^^25^^,^^26^ Zhao et al. demonstrated that in prostate cancer, intrachromosomal rearrangements occur more frequently within topologically associating domains than expected by chance. However, their analysis did not extend to interchromosomal translocations. To investigate whether the chromatin conformation influences the formation of inter-chromosomal translocations in CRPC, we analyzed a published cohort of CRPC structural variants containing 15,526 interchromosomal translocations from 101 CRPC samples.^23^ We compared the observed frequency of compartment-pair combinations to an expected distribution derived from shuffled compartment labels (see STAR Methods). Interchromosomal translocations occurred more likely between A compartments, compared to BB- and AB-combinations (*p* < 0.001) (Figure 1D).

We next examined the contact frequency of translocation sites averaged across BPH samples. These samples typically lack structural variation and therefore reflect baseline chromatin organization without rearrangement-induced artifacts, allowing us to determine whether loci involved in translocations were spatially proximal prior to translocation. Interchromosomal translocations between A compartments showed significantly stronger interactions than those between B compartments or between A- and B compartments (*p* < 2.2e−16, Wilcoxon). To establish a random background, we shuffled translocation breakpoint pairs. Observed AA-translocations exhibited more contacts than randomly generated AA (*P* = 1e−7, Wilcoxon), as well as A/B-translocations compared to randomly generated A/B-pairs (*p* = 1.2e−28, Wilcoxon) (Figure 1E). These findings suggest that translocations are guided by spatial proximity and that A compartments exhibit stronger interchromosomal contacts and are more prone to translocations than B compartments, consistent with their preferential co-localization toward the nuclear interior. In contrast to B compartments, that are thought to localize to the nuclear periphery, remaining more spatially separated from B compartments of other chromosomes.

We next investigated whether structural rearrangements activate oncogenes through enhancer hijacking in prostate cancer. Integrative analysis of Hi-C-derived structural variants (Table S6) revealed formation of “neoloops” in reconstructed breakpoint regions.^27^^,^^28^ We considered all loops detected in the assembled regions as neoloops whether they spanned the break junction or not. Neoloops were more abundant in CRPC than PC and were significantly associated with outlier gene expression in multiple CRPC samples (Figure S5). Although disrupted regions frequently coincided with copy-number alterations, 67% neoloop-associated genes showed expression levels exceeding those expected from copy number alone, indicating additional regulatory mechanisms (Figure S5B). A total of 138 neoloops spanning breakpoint junctions in interchromosomal translocations were detected across all cancer samples. Nearly half of these interactions (47.8%) occurred between A compartments, while 43.5% connected A and B compartments. In contrast, only 8.7% were between B compartments, supporting a model in which enhancer hijacking is largely confined to active genomic regions.

*ETV1* provided a notable example of copy-number-independent activation. Across the RNA-seq cohort, four tumors exhibited high *ETV1* expression. All three expression-outlier samples with Hi-C data harbored ETV1 rearrangements, including recurrent *TMPRSS2–ETV1* and *SLC45A3–ETV1* fusions, as well as a translocation linking *ETV1* to *MIPOL1* (Figure S5).^29^^,^^30^ Reconstruction of the latter event demonstrated contact between the *ETV1* promoter and distal enhancers residing inside *MIPOL1* and normally regulating *FOXA1* expression, confirming enhancer hijacking previously described in LNCaP cells.^27^

### A/B compartment organization is altered in prostate cancer

Overall, the average A/B compartment scores were highly correlated between groups (Pearson’s r = 0.97 for BPH vs. PC and 0.96 for BPH vs. CRPC), seemingly indicating minimal compartment shifting (Figure S6). However, we hypothesized that the signal was attenuated by the non-tumor cellular composition of the tissue samples. Supporting this, the difference in A/B compartment scores between cancer and BPH samples correlated with tumor purity (Spearman correlation of 0.88, *p* = 0.0002; Figure S7). To detect compartment shifts while accounting for tumor purity, we modeled the A/B score as a mixture of cancer and normal prostate cells (see STAR Methods, Figure S8). Using this model, we identified 381 Mb of activating compartments (increased A/B score) and 403 Mb of inactivating compartments (decreased A/B score) between BPH and all cancer samples, applying a q value threshold of 0.05. We divided the genome into six categories: stable A, activating A, inactivating A, stable B, activating B and inactivating B compartments (Table S7). Compartment alterations were generally stable in leave-one-sample-out analysis, although exclusion of PC19403, a high-tumor-purity sample, resulted in fewer significant compartment shifts (Table S8). In particular, activating B compartments showed distinctive interaction patterns in high tumor purity samples consistent with shifting from B compartments toward A compartments (Figure 2A). The extent of compartment shifts was consistent with tumor purity (Figure 2B; Figures S8 and S9) and did not associate with copy number alterations (Figure S10). We estimated A/B scores at 100% tumor purity by fitting a linear model between A/B scores and tumor purity. Complete compartment switches were observed in 66% of activating B compartments (B→A) and 44% of inactivating A compartments (A→B) (Table S7). Consistent with compartment change, activating B compartments had fewer contacts to the nuclear lamin-associated regions in high tumor purity samples compared to BPH (Figures 2C and 2D). This was validated in published cell line data of LNCaP and RWPE1 cell lines, representing prostate cancer and normal basal prostate cell types, respectively (Figure 2E).^8^ Chromatin conformation has been shown to influence mutation rates.^31^ Indeed, mutation rates correlated highly with compartment identity (Figure S11). Activated B compartments were associated with distinct mutation rates consistently across prostate cancers compared to other cancer types in the Pan-Cancer Analysis of Whole Genomes (PCAWG) dataset,^32^ suggesting that these compartment shifts may be widely present in prostate cancer (Figure S11). Consistent with this, analysis of interchromosomal translocations revealed distinct associations between compartment classes, with Activating B compartments showing no depletion for translocations with A compartments, while translocations between stable A and B compartments were significantly depleted (Figure S12). Together, these patterns are consistent with repositioning of activating B compartments toward A compartments in prostate cancer. Only 25 Mb and 24 Mb of the genome showed activating or inactivating changes, respectively, between CRPC and PC. However, limited statistical power may have constrained detection.

**Figure 2:**
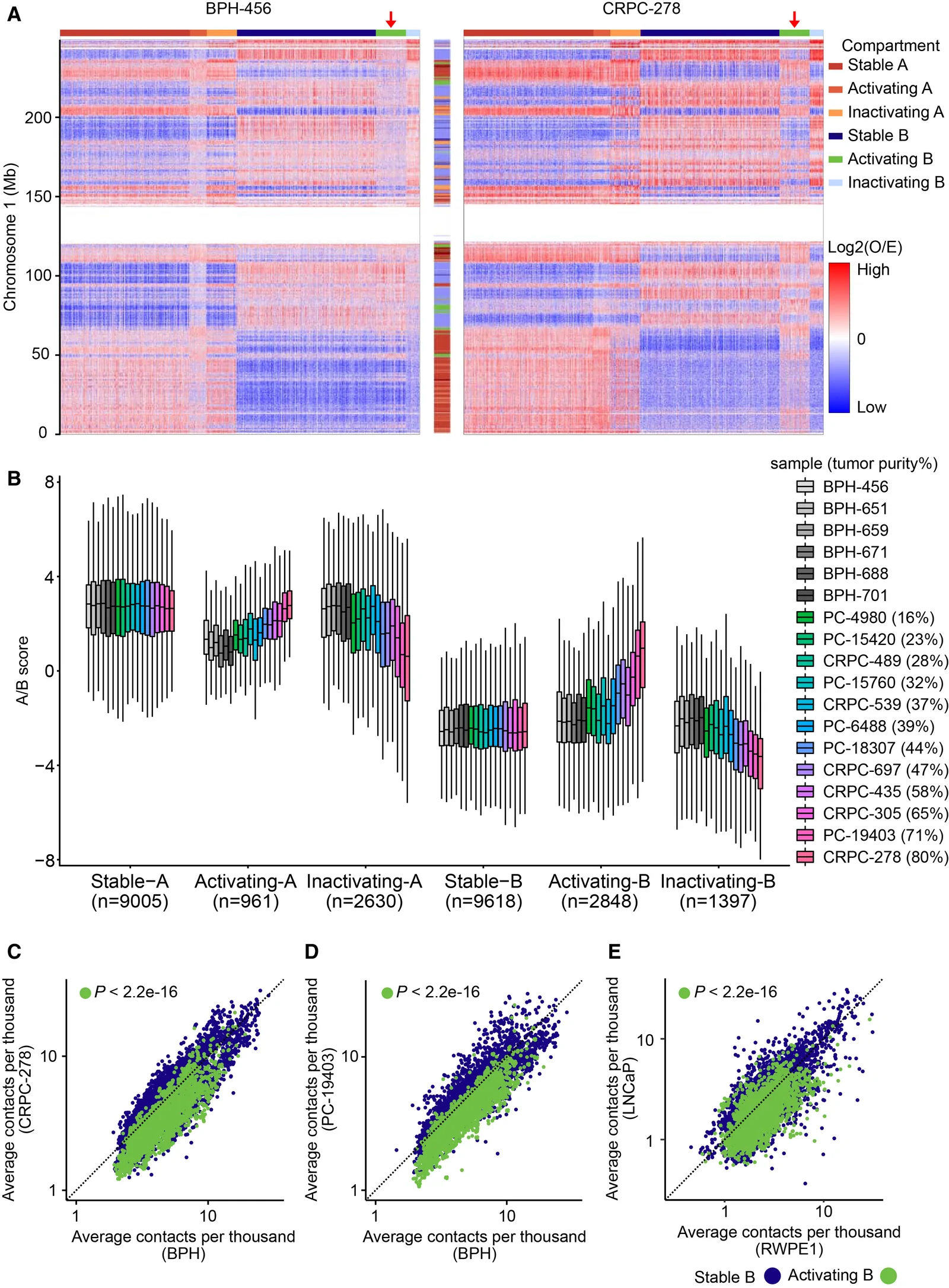
Compartment changes in prostate cancer correlate with tumor purity and are associated with reduced contacts to lamin-associated regions (A) Observed-to-expected (O/E) normalized contact matrices for representative BPH and high-purity CRPC samples. Columns of interaction matrices were shuffled and resorted based on their compartment annotations to reveal the interaction patterns characteristic to each compartment type. Notably, activating B-compartments show distinct interaction patterns consistent with change from B-compartment to-A compartment in the CRPC-278 sample. This is indicated with red arrows. (B) The A/B score in inactivating and activating compartments correlate with tumor purity in cancer samples. Boxplots of the A/B score in 100 kb bins grouped by their compartment annotation. Cancer samples are sorted by their tumor purity in ascending order. Boxplots show the median as the central line, with whiskers extending to 1.5 times the interquartile range (IQR) from the lower and upper quartiles. (C–E) Scatterplots comparing the average number of contacts with lamin-associated regions. (C and D) show comparisons between the average BPH signal and two high tumor purity tumor samples CRPC278 and PC19403, and (E) compares the normal prostate cell line (RWPE1) with the prostate cancer cell line (LNCaP). Each point corresponds to a 100 kb genomic bin annotated as either a stable B-compartment or an activating B-compartment. Contacts within 5 Mb were excluded to remove local (self) interactions. After balancing normalization, contact counts were normalized with total contacts at each 100 kb bin multiplied by a thousand and subsequently quantile-normalized between samples. The dotted line indicates the line of identity (x = y). *p* values are calculated using paired Wilcoxon signed-rank test.

### Activating compartments are enriched for luminal and prostate cancer specific gene expression

Next, we investigated the relationship between gene expression changes and chromatin compartment alterations. Analysis of bulk sequencing data from partially matched samples (Table S1) revealed that genes upregulated in prostate cancer were significantly enriched in activating B compartment compared to the rest of the genome (11.3% vs. 4.4%, *p* = 1.9e−16). No enrichment of differentially expressed genes between CRPC and primary prostate was observed in compartments that differed between CRPC and primary prostate cancer (*p* = 0.67).

To determine whether these compartment-associated expression differences reflected intrinsic cancer-specific changes or simply enrichment of luminal-type cells within tumor samples, we integrated publicly available single-cell transcriptomic datasets from the prostate (98 samples from 65 patients; see STAR Methods).^33^^,^^34^^,^^35^^,^^36^^,^^37^^,^^38^^,^^39^^,^^40^ After merging the datasets, we computationally identified and purified normal luminal, normal basal, and malignant epithelial populations (Figure S13). After quality filtering and annotation, 10392, 6269, and 23830 cells were classified as normal luminal, normal basal, and cancer cells, respectively. For luminal and basal cells, only benign samples were utilized to avoid mixing them with cancer cells.

Differential gene expression analysis was performed using pseudo-bulk profiles after removing low coverage samples (total number of reads in pseudo-bulk less than 50,000). To control for batch effects only datasets containing both normal and malignant samples were included. This resulted in 24 luminal, 28 basal and 26 cancer pseudo bulk samples. Cancer cells exhibited a marked significant decrease (*p* = 2.3e−12, Wilcoxon; Figure 3A) in luminal signature expression and a marginal increase in basal signature expression relative to normal luminal cells (*p* = 0.04, Wilcoxon; Figure 3B), while basal cells showed the strongest divergence in both signatures (Figures 3A and 3B). We identified 3,486 and 1,630 differentially expressed genes between luminal and basal cells, and between luminal and cancer cells, respectively (Figures 3C and 3D). Among the top upregulated genes in cancer cells was *ERG*, a hallmark driver of prostate cancer resulting from the *TMPRSS2–ERG* fusion, confirming the correct annotation of malignant cells. There was no significant overlap between luminal- and cancer-upregulated genes (odds ratio of 0.98, *p* = 0.91), indicating distinct gene expression programs.

**Figure 3:**
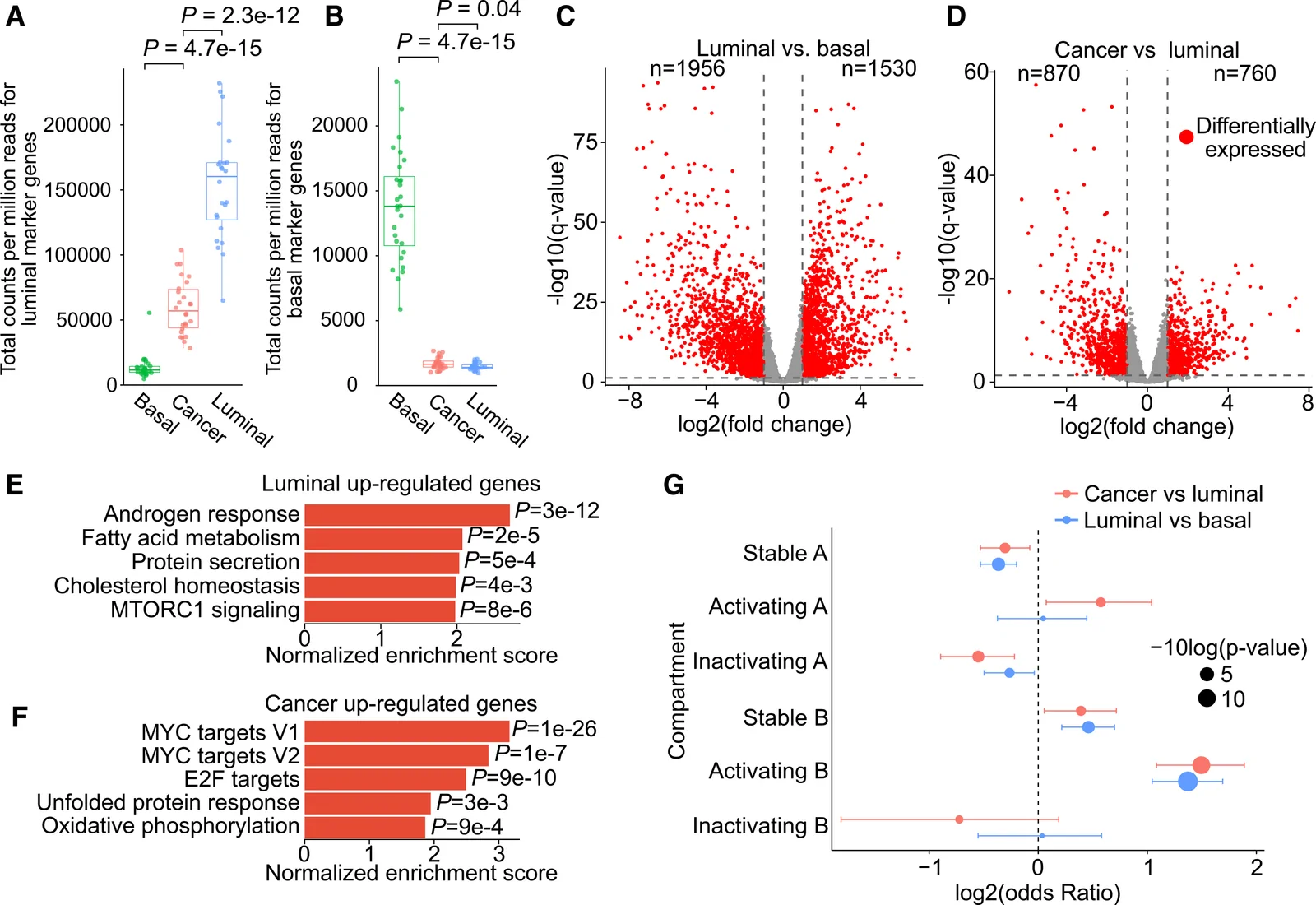
Cancer-upregulated genes are enriched in activating B-compartments (A) Total counts per million reads of luminal marker genes in pseudo bulk samples. (B) Total counts per million reads of basal marker genes in pseudo bulk samples (*n* = 24 luminal, *n* = 28 basal, and *n* = 26 cancer samples). Boxplots show the median as the central line, with whiskers extending to 1.5 times the interquartile range (IQR) from the lower and upper quartiles. *p* values are calculated using the Wilcoxon signed-rank test. (C) Volcano plot of gene expression between luminal and basal single cell pseudo bulk samples. Differentially expressed genes are colored red. Vertical lines indicate log2 fold thresholds of −1 and 1 and horizontal line indicates adjusted *p* value threshold of 0.05. (D) Volcano plot of gene expression between cancer and luminal cells. (E) Gene set enrichment analysis (GSEA) of differentially expressed genes between luminal and basal cells.^41^ (F) GSEA analysis between cancer and luminal cells. *p* values are calculated using an adaptive multi-level split Monte-Carlo scheme implemented in the fgsea package. (G) Forest plot of the enrichment of cancer and luminal up-regulated genes in different genomic compartments. Both cancer- and luminal-up-regulated genes are strongly enriched for activating B-compartments. Point size reflects –log10(*p* value) and error bars denote 95% confidence intervals. *p* values are calculated using the Fisher’s exact test.

Genes upregulated in luminal cells were enriched for androgen receptor (AR) response pathways (*P* = 3e−12, Figure 3E), whereas those upregulated in cancer cells were enriched for MYC target genes (*P* = 1e−26) as well as genes related to cell proliferation and unfolded protein response (Figure 3F). Notably, luminal-upregulated genes were enriched in activating B compartments relative to the rest of the genome (25% and 11.4% of genes upregulated, respectively; *p* = 2.1e−15; Figure 3G), suggesting that at least part of the observed compartmentalization changes may reflect luminal-specific chromatin states. For example, *KLK3* (prostate specific antigen) and the other adjacent KLK-genes are located at activating B compartments, consistent with their luminal specific expression. To address whether differences in luminal cell abundance could account for the observed compartmentalization changes, we used matched RNA-seq data to calculate a luminal cell signature as a proxy for luminal cell abundance in the Hi-C samples. We found no association between the luminal signature score and deviations in A/B compartment scores in BPH samples (Figure S14).

Cancer-upregulated genes were also significantly enriched in these regions compared to rest of the genome (14.7% and 5.7% of genes, respectively; *p* = 1.1e−11 Figure 3G), with 68 cancer-upregulated genes located within activating B compartments (Table S9). Prominent prostate cancer associated genes located in activating B compartments include, for example, *PCA3*, *AKR1C3*, *ONECUT2* and *TWIST1*.^42^^,^^43^^,^^44^^,^^45^ Notably, PCA3 is a prostate-cancer-specific AR-regulated long noncoding RNA that is consistently overexpressed in prostate cancer and is used clinically as a molecular biomarker to aid prostate cancer diagnosis.^45^ Despite the frequent overexpression, no recurrent genomic alteration has been reported in the genomic loci of *PCA3*, suggesting epigenetic activation.^1^ Collectively, these results indicate that the enrichment of cancer-upregulated genes in activating B compartments represents a cancer-specific gene expression signature, distinct from the luminal-specific expression program. However, as previously described, the majority of genes located within shifting compartments were not differentially expressed, indicating that chromatin conformation is only one layer within a broader, multilayered epigenetic regulatory architecture controlling gene expression.^46^^,^^47^^,^^48^

### Activating B compartments are enriched for cancer-specific enhancers and transcription factors associated with the AR-cistrome

We sought to characterize the epigenetic landscape underlying chromatin compartmentalization alterations. Trimethylation of lysine 9 on histone 3 tail (H3K9me3) characterizes repressed heterochromatin and is strongly associated with lamina-associated domains.^49^ Using publicly available H3K9me3 ChIP-seq data from normal and prostate cancer cell lines,^50^ we examined how alterations in A/B compartment status are reflected in heterochromatin organization. Overall, H3K9me3 signal intensity was highly correlated with the A/B compartment score, supporting a close relationship between heterochromatin and nuclear lamina association (Figure S15). Regions that become activated in prostate cancer exhibit high H3K9me3 signal in normal prostate cell line HPrEC, with only a modest reduction in H3K9me3 solely in LNCaP cells but not in VCaP, 22Rv1 and PC3 cancer cell lines. LNCaP, VCaP and 22Rv1represent advanced AR positive and PC3 represents AR-negative prostate cancer. (Figure S15).

To investigate the epigenetic landscape across chromatin compartments, we analyzed a unified set of 85,450 ATAC-seq peaks detected across 38 prostate samples partially matched with our Hi-C cohort (Table S1).^21^ 65% of ATAC-peaks belonged to A compartments and 35% to B compartments. We annotated the ATAC-peaks with epigenetic states derived from prostate cell lines.^51^ Compared to stable B compartments, activating B compartments showed enrichment for active chromatin states and depletion of heterochromatic states in AR expressing cancer cell lines; this enrichment pattern was absent in the normal basal prostate cell line PrEC and in the AR-negative PC3 cell line, which resembles neuroendocrine small-cell prostate carcinoma. This suggests chromatin activation in luminal cells and/or AR-dependent cancer cells. (Figure 4A). We further refined this pattern by defining sets of prostate cancer-specific and normal-specific enhancers. Prostate cancer-specific enhancers were identified as regions annotated as active enhancers in LNCaP and/or VCaP cells but not in normal PrEC cells, whereas normal-specific enhancers were defined as regions active in PrEC cells but not in LNCaP or VCaP cells. ATAC-seq peaks corresponding to these cancer-specific enhancers showed strong enrichment within activating B compartments compared to the rest of the genome (44% vs. 27% of peaks, respectively; *p* = 2.1e−142) while normal specific enhancers were depleted (3% vs. 10% of peaks, respectively; *p* = 3.9e−62).

**Figure 4:**
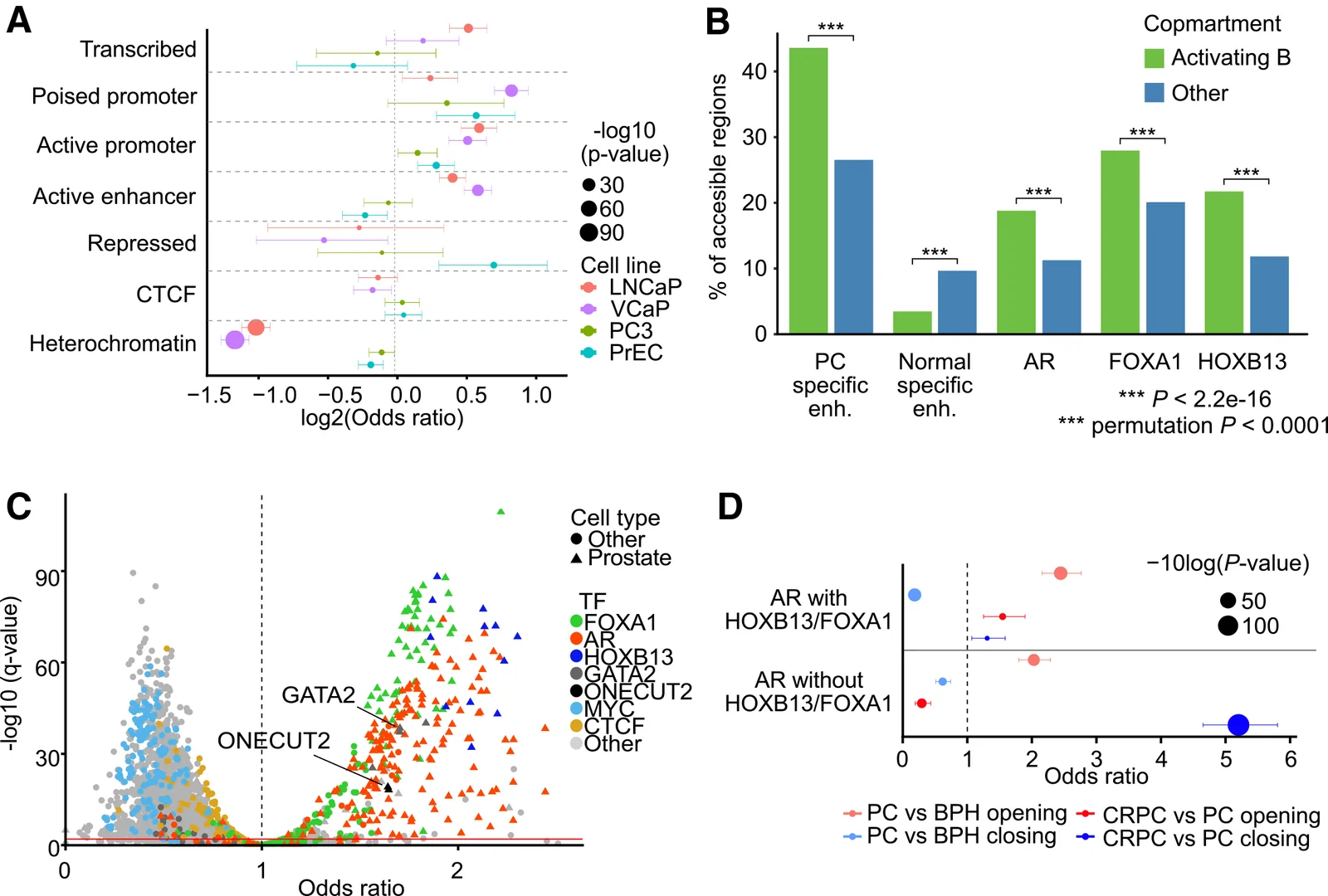
Activating B-compartments are associated with activating epigenetic state in prostate cancer (A) Enrichment analysis of chromatin states in ATAC peaks located in activating B-compartments compared to stable B-compartments shows their enrichment in active states specifically in AR-dependent prostate cancer cells. Chromatin annotations were derived from a normal prostate cell line (PrEC), two AR positive cancer cell lines (LNCaP and VCaP), and one AR negative cell line (PC3).^51^ (B) Accessible regions are enriched for prostate cancer (PC)–specific enhancers and prostate lineage TFs relative to the rest of the genome. Prostate cancer-specific enhancers are defined as peaks annotated as active enhancers in the LNCaP and/or VCaP cell lines, but not in the normal PrEC cell line; conversely, normal-specific enhancers are defined as peaks active in PrEC but not in LNCaP or VCaP. Enrichment *p* values were calculated using both Fisher’s exact test and block permutation of chromatin compartments to account for non-independence due to spatial proximity. (C) Prostate lineage TF binding sites are enriched in ATAC-peaks located in activating B-compartments compared to the rest of the genome. Data points represent individual TF binding experiments from the UNIBIND database. Experiments from prostate cells are indicated as triangles. *p* values are calculated using the Fisher’s exact test and were adjusted for multiple testing using the Benjamini–Hochberg method. (D) Enrichment of AR-FOXA-HOXB13 co-bound sites and AR-only binding sites within regions that are differentially accessible between PC and CRPC. For A and D *p* values are calculated using the Fisher’s exact test. Point size reflects –log10(*p* value) and error bars denote 95% confidence intervals.

Next, we integrated TF binding data from the UniBind database with our ATAC-seq data.^52^ ATAC-peaks located in activating B compartments were markedly enriched for AR, FOXA1, and HOXB13 binding sites—key TFs associated with the luminal prostate lineage and prostate cancer^6^—compared to other genomic regions (Figures 4B and 4C). In addition, binding sites for GATA2 and ONECUT2, TFs with established roles in prostate cancer,^42^^,^^53^ were also enriched (Figure 4C; Table S10). Of these TFs, *ONECUT2* is located in an activating B compartment. To assess whether TF expression was associated with activating B compartments, we fitted a linear model accounting for tumor purity. *HOXB13* expression was moderately associated with A/B compartment shifts toward activating B compartments (FDR = 0.023), explaining an additional 8.0% of the variance beyond tumor purity (Table S11). Notably, enrichment was specific for these prostate lineage TFs, while other TFs (such as MYC) were mainly depleted from activating B compartments relative to the rest of the genome (Figure 4C; Table S10). Most Hi-C samples were ETS fusion-positive, with 10 out of 12 samples harboring ETS fusions, including three ETV1-positive and seven ERG fusion-positive cases (Table S1). Despite previous reports linking ERG to chromatin remodeling,^13^ no enrichment of ETS family TFs was observed in activating B compartments. Collectively these results indicate that activating B compartments undergo epigenetic activation associated with the AR regulatory program. This is highlighted in the *PCA3* gene located in an activating B compartment, where clinical tissue data from external dataset shows cancer specific active histone modifications (H3K27ac and H3K4me3) and AR, FOXA1 and HOXB13 binding (Figure S16).^3^

To investigate how local chromatin conformation is related to compartments, we identified chromatin loops from our Hi-C data using MUSTACHE at 10 kb resolution.^54^ We generated a unified loop set by retaining loops detected in at least two samples (see STAR Methods), resulting in a total of 14,718 loops (Table S12). Consistent with established chromatin biology, 87% of loops contained convergent CTCF motifs at their anchors.^15^

We next performed differential loop analysis between BPH and all cancer samples (see STAR Methods), identifying 699 upregulated and 519 downregulated loops in cancer. A total of 6030 genes evaluated in pseudo bulk analysis overlapped at least one loop anchor (located within 10 kb from the loop anchor). Among these loop-associated genes, both luminal-upregulated genes (9.6% vs. 4.5% of genes, respectively; *p* = 2.4e−7) and cancer-upregulated genes (12.4% vs. 4.6% of genes, respectively; *p* = 7.5e−9) were significantly enriched at upregulated loops compared with other loop-associated genes. Loops were significantly more often upregulated in activating B compartments compared with the rest of the genome (36% vs. 13% of loops, respectively; *p* = 1.6e−50). Consistent with this observation, upregulated loops were also enriched for FOXA1, HOXB13, and AR binding sites (Figure S17). These results suggest that chromatin loops undergo AR-cistrome associated rewiring at activating compartments.

To study whether changes in chromatin accessibility correlated with compartment changes, we developed a pipeline that corrects biases introduced by copy-number alterations and tumor purity to identify differentially accessible peaks from ATAC-seq data. Tumor purity was estimated directly from ATAC-derived copy-number profiles similar to Hi-C (see STAR Methods and Figures S18 and S19), ranging between 12% and 70% (median 24%) in PC and 20%–100% (median 72%) in CRPC (Table S1). After implementing copy number and tumor content aware differential peak calling (see STAR Methods, Figure S20), we identified 4407 opening and 4900 closing peaks between PC and BPH, and 2101 opening and 2672 closing peaks between PC and CRPC. However, differentially accessible regions were not associated with activating B compartments (Figure S21).

We next tested the enrichment of differential peaks across all transcription-factor binding datasets in the UniBind database.^52^ In PC, opening peaks were enriched for AR (25% vs. 11% of all peaks, *p* < *2*.*2e−16*), FOXA1 (43% vs. 20% of all peaks, *p* < *2*.*2e−16*), and HOXB13 (22% vs. 12% of all peaks, *p* < *2*.*2e−16*) binding sites, whereas closing peaks were enriched for TP63 (19% vs. 3% of all peaks, *p* < *2*.*2e−16*), a basal-cell marker—consistent with the luminal phenotype of prostate cancer cells (Figure S22A). In CRPC, closing peaks were enriched for FOXA1 (31% vs. 20%, *p* < *2*.*2e−16*) and AR (32% vs. 11%, *p* < *2*.*2e−16*). Notably, HOXB13 binding sites were not enriched among closing peaks despite their frequent co-binding with AR. Instead, HOXB13 sites were enriched among peaks opening in CRPC (23% vs. 12%, *p* < *2*.*2e−16*) (Figure S22B).

To further resolve these patterns, we partitioned AR sites into two groups: those co-bound by both FOXA1 and HOXB13 (AR–HOXB13–FOXA1) and those bound by neither (“other AR binding sites”). Regions that lost accessibility in CRPC were strongly enriched for the other AR binding sites, whereas regions opening in CRPC were depleted of them (Figure 4D). These findings suggest that CRPC preserves the canonical AR–FOXA1–HOXB13 regulatory program while selectively silencing an alternative AR-associated regulatory program.

## Discussion

We present a clinical cohort of Hi-C sequencing data from prostate tissue samples spanning different states of the disease trajectory, from a non-malignant state (modeled by BPH) to the emergence of castration resistance. Previous studies have been limited to advanced tumors or cell lines or have not explicitly addressed the heterogeneous cell content of clinical samples leaving *in vivo* chromatin compartment dynamics across prostate cancer progression largely unexplored.^8^^,^^9^^,^^10^^,^^11^^,^^12^^,^^13^ To address these gaps, we assembled a cohort covering the full spectrum of prostate cancer and developed an analysis framework that rigorously accounts for tumor purity. We show that specific genomic regions in prostate cancer undergo compartment shifting from B toward A compartments. These regions are linked to luminal and prostate cancer-specific transcriptional programs and are characterized by an activating epigenetic landscape, enrichment for AR cistrome associated TF binding, and the emergence of cancer-specific chromatin loops. Notably, this regulatory configuration is also observed in CRPC, suggesting that these compartment shifts persist into the castration-resistant state. Primary prostate cancer is characterized by a relatively low rate of genomic alterations,^1^ yet exhibits extensive transcriptomic reprogramming. Our results suggest that chromatin compartment reorganization may contribute to changes in epigenetic regulation and gene expression programs in prostate cancer.

TFs associated with the luminal prostate phenotype, including AR, FOXA1, and HOXB13, were enriched at activating compartment shifts. These factors frequently co-bind and serve as key regulators of luminal identity and prostate carcinogenesis. FOXA1, a pioneer factor, can bind to condensed chromatin and facilitate recruitment of additional regulators. Notably, members of the FOXA family have been implicated in chromatin-lamina reorganization in other contexts; in aged hepatocytes, regions that dissociate from the nuclear lamina are enriched for forkhead motifs and FOXA2 binding sites.^55^ Given the high sequence homology between FOXA1 and FOXA2 in their forkhead DNA-binding domains and their substantial overlap in genomic occupancy,^56^ it is plausible that FOXA1 contributes similarly to lamina-dissociation in prostate cancer cells. Furthermore, recent evidence suggests that AR may influence nuclear lamina structure by directly interacting with lamin A/C. AR loss in cancer-associated fibroblasts leads to lamin A/C phosphorylation, nuclear membrane abnormalities, and rupture.^57^ Nevertheless, the broader spatial relationship between AR binding sites and nuclear lamina association in prostate cancer remains unclear. Mechanistic studies could help elucidate the role of the AR–FOXA1–HOXB13 axis in shaping chromatin compartmentalization. Notably, ERG has been implicated in the reorganization of the three-dimensional genome in normal prostate cell lines, establishing that TFs can modulate chromatin conformation in prostate cells.^13^ However, ten of the twelve tumors analyzed here were ETS fusion-positive, but we did not observe enrichment of ERG or ETV1 binding at prostate cancer-specific activating regions.

Despite these insights, the mechanisms controlling chromatin compartmentalization and selective association with the nuclear lamina remain poorly understood. Our results are descriptive and do not resolve causality—whether spatial reorganization precedes epigenetic activation or vice versa, or whether the identified prostate lineage TFs contribute to compartment shifting. These compartment shifts appear to be a common feature of prostate cancer, as supported by external gene expression and mutation datasets that correlate strongly with compartment identity. It is unlikely that these shifts are driven by a single genetic event, as primary prostate cancer lacks widely recurrent driver mutations beyond the ETS-family fusions.

In conclusion, our work establishes that higher order chromatin conformation is tightly coupled to cancer-specific genomic alterations, gene expression and epigenetic reprogramming. We show that dysregulation of higher order chromatin is potentially recurrent across prostate cancers. Overall, our findings provide new insights into the mechanisms of epigenomic rewiring in prostate cancer and advance our understanding of how higher order chromatin organization may contribute to carcinogenesis of the prostate.

### Limitations of the study

Our study is constrained by a relatively small sample size and the lower tumor purity of primary prostate cancer samples compared to CRPC samples, which reduces our power to detect alterations associated with castration resistance. Additionally, we could not resolve the cellular composition of BPH samples. As BPH is not characterized by a clonal expansion of a single cell type but instead represents a complex, multicellular process involving epithelial, stromal, and inflammatory components, the use of bulk sequencing data likely obscures cell type-specific signals. Consequently, we cannot fully disentangle whether the observed molecular signals are truly cancer-specific or instead reflect enrichment of luminal epithelial cells or other non-malignant cellular compartments.

## Resource availability

### Lead contact

Requests for further information and resources should be directed to and will be fulfilled by the lead contact, Matti Nykter (matti.nykter@tuni.fi).

### Materials availability

This study did not generate new unique reagents.

### Data and code availability

• The raw sequencing data are available from the authors, although certain restrictions apply to their access. These data may be shared with qualified researchers, provided their use aligns with the conditions set by ethical approvals and informed consent for research on prostatic diseases. All data handling must comply with GDPR and other applicable data protection regulations and requires the completion of a material transfer agreement with the relevant data controllers. Processed data files for Hi-C and ATAC-seq data are deposited to Gene Expression Omnibus under accession no GSE328401 and GSE328397, respectively.
• The code used for the analyses presented in this study is publicly available on GitHub (https://github.com/tuomovirtanen/Tampere-Hi-C) and archived on Zenodo at https://doi.org/10.5281/zenodo.22642506.

## Acknowledgments

We thank all the patients who generously provided samples for this study. Their participation was essential to make this research possible. We are grateful for their contribution and trust. Päivi Martikainen and Hanna Selin are recognized for technical assistance in laboratory work. We also acknowledge the PCAWG Consortium for making their data available and for their efforts in generating and curating the dataset. The study was financially supported by the 10.13039/501100002341Research Council of Finland (#352818 to M.N.), 10.13039/501100010711Cancer Foundation Finland (M.N., A.U., T.V.); 10.13039/501100006306Sigrid Jusélius Foundation (M.N., K.J.R.); and Competitive State Research Financing of the Expert Responsibility area of 10.13039/501100010600Tampere University Hospital (M.N., K.J.R., A.U.). A.U. wished to thank also the 10.13039/501100002341Research Council of Finland project no. 349314; 10.13039/100008730Norwegian Cancer Society project no. 273672–2023 and the 10.13039/100005235Tampere Institute for Advanced Study, Finland. T.V. has received funding also from 10.13039/501100003125Finnish Cultural Foundation. E.A. has received postdoctoral research funding from the 10.13039/501100003125Finnish Cultural Foundation.

## Author contributions

T. Virtanen performed formal analysis, data analysis, and wrote the manuscript as the first author. E.A. contributed to supporting analyses of ATAC-seq and participated in manuscript review and editing. R.N. and J.U.-M. performed experimental work and generated Hi-C and ATAC-seq data, respectively. A.K. contributed supporting analysis of single-cell data, and J.S. performed supporting analysis of tumor purity estimation. F.T. contributed to supporting analyses of RNA-seq data and participated in manuscript review and editing. T.T., T.J.M., and T. Visakorpi contributed to sample collection. J.K., A.U., and K.R. contributed to scientific guidance, manuscript review and editing. Experimental work was supervised by K.R. The work was initiated and supervised by M.N. All authors have reviewed and accepted the manuscript.

## Declaration of interests

TJ Murtola: Lecture fees from Astellas, Bayer, Johnson & Johnson, and Pfizer; paid consultant for Astellas, AstraZeneca, Bayer, Johnson & Johnson, Pfizer, MSD and Accord; clinical trial funding from AstraZeneca, Bayer, Pfizer, Johnson & Johnson and MSD.

## Declaration of generative AI and AI-assisted technologies in the manuscript preparation process

During the preparation of this work, the authors used OpenAI ChatGPT GPT-5.5 in order to improve the language. After using this tool/service, the authors reviewed and edited the content as needed and take full responsibility for the content of the published article.

## STAR★Methods

### Key resources table

**Table:** 

REAGENT or RESOURCE	SOURCE	IDENTIFIER
**Critical commercial assays**		

Arima-HiC Kit	Arima Genomics, San Diego, CA, USA	Cat#A510008
Kapa Hyper Prep Kit with Library Amplification Module	Roche, Basel, Switzerland	Cat#KK8500
cOmplete Protease Inhibitor Cocktail	Roche, Basel, Switzerland	Cat#4693132001
MinElute PCR Purification Kit	QIAGEN	Cat#28006
Agencourt AMPure XP beads	Beckman Coulter	Cat#A63881
Tn5 Transposase	Illumina	Cat#FC-121-1030

**Deposited data**		

Hi-C	This publication	GEO: GSE328401
ATAC-seq	This publication	GEO: GSE328397
RNA-seq	Latonen et al.^22^	European Genome-Phenome Archive: EGAS00001000526
cell line Hi-C data	Rhie et al.^8^	GEO: GSE118629
PCAWG somatic mutation data	ICGC/TCGA PCAWG Consortium^32^	December 13, 2023
Lamin-B1 ChIP-seq data	Shah et al.^24^	GEO: GSE155244
CRPC structural-variation data	Quigley et al.^23^	
Single cell data	Kiviaho et al.^40^	
H3K9me3 ChIP-seq data	Guo et al.^50^	GEO: GSE208449
UniBind TF-binding data	Puig et al.^52^	https://unibind.uio.no/
Clinical prostate ChIP-seq data	Pomerantz et al.^3^	GEO: GSE130408

**Software and algorithms**		

Arima Genomics Hi-C Mapping Pipeline	Arima Genomics, https://github.com/arimagenomics/mapping_pipeline	Oct 2021
BWA-MEM	Li et al.^58^	v0.7.17
SAMtools	Li et al.^59^	v1.8; v1.19.2
Juicer Tools	Durand et al.^60^	v1.14.08
HiCRep	Yang et al.^61^	V 1.12.2
Picard Toolkit	Broad Institute, https://github.com/broadinstitute/picard	v2.18.22; v3.0.0
FastQC	Babraham Bioinformatics, https://github.com/s-andrews/fastqc	v0.11.7
Trim Galore	Babraham Bioinformatics, https://github.com/felixkrueger/trimgalore	v0.6.10
Bowtie2	Langmead et al.^62^	v2.5.1
BEDTools	Quinlan and Hall^63^	v2.31.1
MACS3	Zhang et al.^64^	v3.0.1
ATAC Iterative Overlap Peak Merging (createIterativeOverlapPeakSet.R)	Corces et al.^65^	https://github.com/corceslab/ATAC_IterativeOverlapPeakMerging/tree/main
featureCounts	Liao et al.^66^	v2.1.1
limma	Ritchie et al.^67^	v3.62.1
edgeR	Robinson et al.^68^	v4.4.2
DESeq2	Love et al.^69^	v1.20
STAR	Dobin et al.^70^	v2.5.3a
MUSTACHE	Ardakany et al.^54^	https://github.com/ay-lab/mustache
pgltools	Greenwal et al.^71^	v2.2.0
Breakfinder	Song et al.^28^	https://github.com/dixonlab/hic_breakfinder
NeoLoopFinder	Wang et al.^27^	https://github.com/XiaoTaoWang/NeoLoopFinder
fgsea	Korotkevich et al.^72^	v1.32
DNAcopy	Olshen et al.^73^	v1.80.0

### Experimental model and study participant details

#### Samples

Fresh-frozen tissue material was sourced from Tampere University Hospital (Tampere, Finland). All tissue samples were obtained from male patients. Given the prostate-specific focus of the study, only male patients were included. The influence of sex on the results was therefore not assessed. Prostate cancer specimens were obtained at the time of radical prostatectomy, while locally recurrent CRPC samples were collected via transurethral resection of the prostate (TURP). BPH samples were acquired using either procedure (Table S1). All samples were snap-frozen and preserved in liquid nitrogen. Histopathological assessment and Gleason grading were carried out by a board-certified pathologist using hematoxylin/eosin-stained sections. Ethical approval for use of the clinical material was granted by the Tampere University Hospital ethics committee, and written informed consent was obtained from all donors. Age of the patient was not available for CRPC and BPH patients. PC patients were aged between 47-71 Years (Table S1). All patients were Finnish.

Chromatin conformation was studied in six BPH, six PC, and six CRPC samples. A total of six samples were analyzed twice as biological replicates (BPH-701, BPH-651, CRPC-305, CRPC-489, CRPC-278, CRPC-435) and one sample was analyzed twice as a technical replicate (CRPC-539).

### Method details

#### Hi-C sequencing

Hi-C libraries were generated using the Arima Hi-C kit (#A510008) (Arima Genomics, San Diego, CA) and KAPA Hyper Prep Kit (Roche, Basel, Switzerland) according to Arima Genomics’s instructions. Freshly frozen tissue samples were sectioned, pulverized and crosslinked according to Arima Genomics’s low input crosslinking protocol (A160132v01, Oct 2019). The DNA digestion with 4-base cutter enzyme cocktail, biotinylation, and in-nucleus ligation to produce proximally ligated DNA were conducted according to Arima Genomics’s User guide for Animal Tissues (A160132v01, Oct 2019). DNA was fragmented using the Covaris S220 system (Covaris, Inc., Woburn, MA) and sequencing libraries were generated using KAPA Hyper Prep Kit (Roche) according to the Arima Genomics’s instructions (A160139v00, Nov 2018). Libraries underwent QC with the 5200 Fragment Analyzer (Advanced Analytical Technologies Inc., Heidelberg, Germany) or the 4150 TapeStation system (Agilent, Santa Clara, CA) before sequencing with Illumina PE150 (600M raw reads) at Novogene (Cambridge, United Kingdom).

#### Tissue sample processing for ATAC-seq

ATAC-seq was performed for 38 samples: 11 BPH, 16 untreated PC, and 11 CRPC samples (Table S1) as part of our previous work.^21^ Frozen tissue blocks were sectioned into two 50 μm slices. Nuclei were isolated from these sections, with all steps performed on ice. Briefly, 6 ml of ice-cold lysis buffer (10 mM Tris-Cl pH 7.4, 10 mM NaCl, 3 mM MgCl_2_, 0.1% (v/v) Igepal CA-630, 1× protease inhibitors (Roche cOmplete)) was added to a pre-cooled Petri dish. Tissue sections were transferred from the tube into the dish using 1 ml of lysis buffer and subsequently minced into smaller fragments with a scalpel. The lysate and tissue fragments were transferred to a 15 ml Falcon tube and passed through a 16 G needle 15 times. Remaining large debris was allowed to settle, and the supernatant was transferred to a fresh tube and centrifuged at 700 × g for 10 minutes at 4°C. The supernatant was discarded, and the pellet was dissolved in a PBS buffer. Nuclei were counted, and 50,000 nuclei were transferred to a new tube, pelleted again at 700 × g for 10 minutes at 4°C, and the supernatant was removed.

#### ATAC-seq library generation and sequencing

ATAC-seq libraries were prepared as previously described.^74^ Briefly, a transposition reaction mix (25 μl 2× TD buffer, 2.5 μl transposase [Tn5; final concentration 100 nM], and 22.5 μl water) was added to the nuclear pellet. The reaction was incubated at 37°C for 45 minutes, followed by PCR amplification. Libraries were purified using the Qiagen MinElute PCR Purification Kit and further cleaned with Agencourt AMPure XP magnetic beads. Sequencing was performed on an Illumina NextSeq platform using high-output 2 × 75 bp settings, with seven samples multiplexed per run.

#### Alignment and Hi-C data preprocessing

Hi-C read processing and alignment were performed following the Arima Genomics mapping pipeline (https://github.com/ArimaGenomics/mapping_pipeline, Oct 2021). Briefly, the paired-end reads were aligned independently using the BWA-MEM algorithm implemented in *BWA v0.7.17*.^58^ Resulting alignments were converted to BAM format using *samtools v1.8*.^59^ To retain only a single mapping position per read, the 5′ mapping was preserved using the *filter_five_end.pl* script from the Arima pipeline. The resulting BAM files were then combined and filtered using the *two_read_bam_combiner.pl* script. Only read pairs with a high mapping quality (MAPQ > 30) were retained.

We utilized HiCRep v1.12.2 to assess concordance between replicates.^61^ Chromosome 1 contact matrices were compared at 100-kb resolution using KR normalization, with a smoothing parameter of 5 and an interaction distance range of 0–5 Mb. After confirming high replicate concordance (Table S2), replicates were merged.

For replicate merging, read groups were assigned with the Picard toolkit v2.18.22, after which BAM files were merged using Picard *MergeSamFiles*. PCR duplicates were subsequently marked and removed using Picard *MarkDuplicates* (https://github.com/broadinstitute/picard). The final BAM files were converted to the Juicer-compatible text format, and .*hic* files were generated with the *pre*-command of *Juicer Tools v1.14.08*.^60^ Published Hi-C data utilized in this research was downloaded from GEO (accession number GSE118629) and analyzed with the same pipeline.^8^

#### ATAC-seq data quality control, alignment, and peak detection

Raw sequencing reads were inspected using fastqc version 0.11.7 (https://www.bioinformatics.babraham.ac.uk/projects/fastqc/) and subsequently trimmed with Trim Galore version 0.6.10 (https://github.com/FelixKrueger/TrimGalore) using parameters --paired --length 20 -q 20. Sequence alignment was performed using Bowtie2 version 2.5.1 against GRCh38 reference genome.^62^ During alignment, parameters --sensitive-local and -X 2000 were used. Sorting and indexing was done with Samtools version 1.19.2.^59^ Finally duplicates were marked using Picard Markduplicates tool version 3.0.0 (http://broadinstitute.github.io/picard/), with parameters VALIDATION_STRINGENCY=LENIENT and REMOVE_DUPLICATES=FALSE. Prior to peak calling, using samtools, BAM files were filtered by removing duplicated reads, secondary alignments, supplementary alignments, unmapped reads (i.e., -F 3332), as well as reads with MAPQ < 20 (i.e., -q 20), and then converted BAM to BED format using bedtools version v2.31.1.^63^ Peak calling was done with MACS3 v3.0.1 using parameters -g hs –shift -75 –extsize 150 -q 0.05 -f BED --nomodel –nolambda --call-summits –keep-dup all.^64^

#### Consensus peak set

To create the consensus peaks set, we used the createIterativeOverlapPeakSet.R script (https://github.com/corceslab/ATAC_IterativeOverlapPeakMerging/tree/main), as described in^65^ using the following parameters: --genome hg38 --spm 5 --rule “2” --extend 250. In addition, to exclude the artefact regions, we merged the ENCODE exclusion set (ENCFF356LFX, https://www.encodeproject.org/files/ENCFF356LFX/) as well as the hg38 human exclusion list (http://mitra.stanford.edu/kundaje/akundaje/release/blacklists/hg38-human/hg38.blacklist.bed.gz) and provided this as an input to the script above using –blacklist parameter.

#### Filtering of sample collection method associated regions

To quantify ATAC-seq signals in consensus peaks, we estimated the local background. Bedtools coverage was used to count the number of overlapping fragments with genome-wide overlapping windows of size 500 bp and steps of 250 bp. To estimate the background b for a given window at position x, we used the following formula: b(x) = max(Q_1(P_10kbp(x)), Q_1(P_100kbp(x)), Q_1(P_chr(x))).

where P10kbp(), P100kbp(), and Pchr() are lists of read counts for all windows within the range of +/-5kbp, +/-50kbp, and chromosome arm, respectively, from position x, excluding the window at position x. Q1 is the value corresponding to the first quartile.

Next, we created a matrix of read counts for the consensus peaks by using the featureCounts tool (v2.1.1) over the BAM files with duplicate reads marked,^66^ using the following parameters: -f -s 0 -Q 20, –primmary, –ignoreDup --countReadPairs --minOverlap 1. Next, we subtracted the mean of estimated backgrounds rounded to the nearest integer in windows that overlapped with each consensus peak to get the background-corrected counts.

We used the limma-voom R package to call the differentially accessible peaks (DAPs) using the background-corrected counts.^67^ Lowly-accessible consensus peaks were first filtered using edgeR::filterByExpr function using min.count = 10, min.prop = 0.7 parameters. Prior to fitting the linear model to each peak using limma::lmFit function, samples were assigned sample-specific quality weights using limma::voomWithQualityWeights function. In addition, via this function data was normalized to log2 counts per million and quantile normalized across samples. Next, to compensate for potential bias due to sample collection procedure (radical prostatectomy=RP and transurethral resection of the prostate=TURP), we first identified consensus peaks that may be affected by sample collection procedure by running the limma-voom only on BPH samples based on their collection procedure (TURP *n*=4, RP *n*=7, using fc=1.2, and FDR=0.2). These peaks were discarded from the consensus peak set. This resulted in 89694 consensus peaks.

#### Quantification of gene expression

Previously published transcriptome sequencing data, including 12 BPH, 30 PC and 13 CRPC samples were utilized.^75^ Reads were aligned to GRCh38 and quantified by STAR version 2.5.3a using Ensembl version 90 annotations.^70^ We obtained quantification of 58243 genes. Lower quartile value of expression distribution across all the samples was used as a threshold to remove low expressed genes, resulting in 18537 protein coding genes (mitochondrial excluded) available for the analysis. The DESeq2 version 1.20 Bioconductor package was used to model the data and extract differentially expressed genes.^69^ We fit the model taking into account both RNA isolation methods (Qiagen™ Trizol™ and Qiagen™ All Prep™) and stages of prostate cancer progression (BPH, PC, CRPC). To address the bias introduced by different extraction protocol, we used coefficients estimated from the model: we extracted coefficients for the RNA isolation method covariate using *DESeq2 coef* function and subtracted these values from library size corrected read counts of Trizol-treated samples in log2 scale. We detected differential expression in two comparisons, BPH to PC and PC to CRPC. A gene was considered differentially expressed (DE) if the absolute median difference of normalized read counts between the groups was greater than 180, the log2 fold change was greater than 1 and the FDR corrected *p*-value lower than 0.05. In the BPH to PC comparison and PC to CRPC comparisons 933 and 533 DE genes were detected.

#### Tumor purity estimation and copy number variation detection from Hi-C data

Tumor purity was estimated from coverage profiles acquired directly from Hi-C data. For each 100kb bin, coverage was calculated from intra chromosomal Hi-C-matrix. The coverage of each sample was normalized by dividing them with their median coverage. After confirming that copynumber variation was absent from BPH-samples they were used as normal control for calculating log2 coverage ratios. We estimated tumor purities and ploidies using a copy number-based methodology described in Herberts et al. 2022^76^ (Figure S1).

For two samples (PC-4980 and PC-6488), we were unable to estimate tumor fraction from genome wide copy number profiles. Both samples had TMPRSS2-ERG fusion and the tumor purity was estimated from the log2 fold ratio of the deletion between TMPRSS2 and ERG, assuming copy number of 1 in the deleted region and otherwise diploid genome (Figures S1B and S1C).

#### Correction of copy number variation artefact from AB-compartments

Copy number variation (CNV) can cause artifacts in detection of A/B-compartments (Figure S2). Large-scale copy number alterations such as whole chromosome arm gains may cause the principal component analysis to detect the copynumber state as the dimension explaining the most variance instead of the underlying compartment structure.^77^ Similarly, in normal un-altered chromosomes the chromosome arm may explain the majority of the variance (so called chromosome arm artefact).^78^ The Hi-C matrices at 100kb resolution were normalized as follows. Copy number segments were detected using the *pcf* function with gamma value of 1000 from the *copynumber* package in R.^79^ Note that, in addition to copy number breakpoints, breaks are introduced in centromeres by the *pcf* function. We partitioned the observed/expected normalized interaction matrices using copy-number segmentation boundaries along both axes. This results in a grid overlay on the interaction matrix, where each grid cell corresponds to a normalization block defined by the pair of interacting copy-number segments (Figure S2). For each block we defined a scaling factor based on the average observed/expected contact ratio in the balance-normalized Hi-C-matrix and divided all the interactions within that block with this scaling factor. Blocks with at least one side smaller than 300kb (3 bins at 100kb resolution) were excluded from correction. These normalized matrices were converted to *.hic* files and once more normalized using the balance method from juicer. The first principal component of the correlation matrix was calculated using the eigenvector command from juicer tools.^60^ As the direction of eigenvectors is arbitrary the eigenvectors were flipped for each chromosome so that gene density was higher in positive values (A-compartments). Results were visually inspected and confirmed that no large-scale copy number or chromosome arm artifacts were present. The chromosome X was removed due to extensive copy number alterations in the *AR* gene in CRPC samples.

#### Identification of consensus lamin binding sites and quantifying lamin association

Published lamin datasets from twelve cell types were used to define consensus lamin-binding sites.^24^ The genome was segmented into 100kb bins, which were overlapped with the T1 lamin state annotations from each cell type as defined by Shah et al. A 100kb bin was classified as a consensus lamin-binding site if it was annotated as T1 lamin in at least ten of the twelve cell types.

For each 100kb bin in each sample, we quantified the total number of intrachromosomal interactions with consensus lamin sites by summing all normalized contacts involving lamin sites and dividing this value by the total number of intrachromosomal contacts for a given bin. The resulting ratio was multiplied by 1000 to obtain contacts per thousand reads.

To compare interactions between samples and differential compartments, we quantified interactions with lamin sites using a similar approach. In this analysis, self-interactions (contacts closer than 5 Mb) were masked, as the majority of reads arise from short-range contacts, which would dominate the signal. Because the lamin consensus annotations are fixed and do not reflect dynamic changes in compartmentalization in our samples, these short contacts would obscure differences between samples. Most consensus lamin sites are stable across conditions; therefore, we used long-range interactions (i.e., contacts to other lamin sites) as a proxy for lamin association. To ensure comparable distributions across samples, lamin interaction values were quantile normalized.

#### Synthetic Hi-C mixture analysis

To validate the linear dilution of A/B-compartment scores caused by tumor purity, we generated synthetic Hi-C mixtures of BPH456 and CRPC278, a high-tumor-purity sample, at defined CRPC278 fractions ranging from 10% to 100%. For each mixture, Hi-C contacts were randomly subsampled from the two source datasets and combined to a total of 500 million contacts, followed by calculation of A/B-compartment PC1 scores using the same analysis pipeline as for the primary samples. PC1 differences relative to BPH456 were compared with those observed in the 100% CRPC278 sample, and robust linear regression was used to assess agreement between the observed and theoretically expected dilution of compartment scores.

#### Detection of differential compartments

Differential compartments were identified from CNV-corrected PC1 values using the *limma v*3.62.1 R-package. To account for variable tumor purity across samples, we explicitly modeled each tumor sample as a mixture of cancer and benign (BPH-like) components. Tumor purity estimates were incorporated directly into the design matrix such that the cancer-associated signal was weighted by the estimated tumor fraction, while the remaining fraction represented the benign component. Specifically, PC and CRPC samples contributed proportionally to both cancer and benign components according to their estimated purity, whereas BPH samples were modeled as purely benign. A linear model without an intercept was fitted to the data, with separate coefficients representing BPH (benign), PC, and CRPC compartments. Empirical Bayes moderation was applied using the *eBayes* function. To improve statistical power both PC and CRPC samples were considered when comparing differences between BPH and cancer samples. Differential compartments were also estimated between PC and CRPC. *p*-values were corrected using the Benjamini–Hochberg method and adjusted *p*-values < 0.05 were considered significant.

#### Single cell RNA-seq analysis

Seven published single-cell RNA-seq datasets from benign and malignant prostate tissue were integrated as described by Kiviaho et al.^33^^,^^34^^,^^35^^,^^36^^,^^37^^,^^38^^,^^39^^,^^40^ Briefly, datasets were processed individually using *scanpy v 1.9.1*. Cells with fewer than 600 UMIs, fewer than 300 detected genes, or more than 20% mitochondrial transcripts were removed; genes with fewer than 10 total counts were excluded. Doublets were removed using *scrublet v0.2.3*. Datasets were integrated using scIB (*scip* v1.1.1). Integration was performed with scVI (*scvitools v0.1.16.1*) with default parameters using 2000 batch-aware highly variable genes. Cells were clustered using Leiden clustering and visualized using UMAP.^80^ Clusters with ≥80% of cells from a single sample were excluded (16 clusters, 42,817 cells). Cluster annotation was performed using marker gene sets as described by Kiviaho et al.^40^

Epithelial clusters were retained for downstream analyses and further annotated using marker gene sets for luminal and basal cells.^40^ Based on the marker gene set expression the clusters were annotated as follows: normal luminal (cluster 5) and basal (clusters 9, 10, 16, 23) clusters were pseudo-bulked using cells from benign samples only, while cancer luminal clusters (clusters with low contribution from normal samples: 12, 13, 27, 28, 37) were pseudo-bulked using cells originating from cancer samples. Differential expression analysis was performed with *edgeR* v4.4.2.^68^ The dataset of origin was used as a covariate to control for batch effects, thus only datasets containing both benign and malignant samples (28 benign samples and 35 cancer samples) were included for differential expression analysis. Pseudo bulk samples with fewer than 50000 transcripts were removed. Resulting in 24 normal luminal samples, 28 normal basal samples and 26 cancer samples. Genes with adjusted *p*-value < 0.05 and |log2 fold change| > 1 were considered as significant. Transcription start sites (TSS) for genes were retrieved from *biomaRt v2.62.0* database and the gene was assigned to a compartment corresponding to the location of the TSS. Gene set enrichment analysis was performed using the *fgsea v1.32.0* R-package.^72^

#### Copy number and tumor purity estimation from ATAC-seq data

The genome was divided into non-overlapping 100 kb bins. Consensus ATAC-seq peaks were extended by 500 bp in both directions to ensure complete exclusion of accessible chromatin regions. These extended peak regions were then masked from the 100 kb bins so that read-depth was quantified from regions outside the ATAC-peaks. Aligned reads were filtered to retain only high-quality (MAPQ ≥ 30) and properly paired fragments. Coverage across the masked bins was quantified using *samtools view* and *bedtools coverage*.

Bin counts for each sample were first normalized by dividing each bin with the sample-specific median count. BPH samples were used as controls, and a reference profile was constructed by taking the median normalized value of each bin across all BPH samples. Log ratios were then calculated as the base-2 logarithm of the normalized bin value divided by the corresponding reference value.

To correct for GC-content bias, a robust local regression was fitted for each sample using the *locfit* v1.5-9.10 package, with normalized log-ratio values as the response and GC fraction of each masked bin as the predictor. The regression used a nearest-neighbor smoothing parameter of 0.3, linear fitting (degree 1), a maximum of 500 knots, and robust fitting to reduce the influence of outliers. The fitted estimates were then subtracted from the raw log2 ratios to obtain GC-corrected log-ratio profiles.

Tumor purity and ploidy were estimated using the same approach that was described for Hi-C data. For downstream copy-number analysis, GC-corrected log-ratio data were segmented using the *segment* function from the *DNAcopy v1.80.0* package.^73^ Segmentation was performed with a significance level of 0.01, standard-deviation–based undoing of splits, an undo threshold of four standard deviations, and a minimum segment width of two bins.

#### Differential peak analysis

Differential accessibility of ATAC-seq peaks was assessed while accounting for copy-number variation, GC-content bias, TSS enrichment, and tumor purity. Peaks overlapping blacklisted regions (Table S5) were excluded from the analysis. Low coverage peaks were removed using the *filterByExpr* function from the edgeR package with default parameters. For each peak, coverage log2 ratios were calculated by overlapping the peak regions with copy-number segments obtained as described above. Counts per million were extracted in log2 scale using the *cpm* function from edgeR, and log2 ratios for each sample were obtained by subtracting the median value of BPH samples. A robust local regression was then fitted per sample using the *locfit v1.5-9.10* package, with the log2-transformed ATAC fold-change as the response and both the coverage log ratio and GC fraction of the peak as predictors. This regression used a nearest-neighbor smoothing parameter of 0.3, linear fitting (degree 1), a maximum of 500 knots, and robust fitting. The fitted values from this regression were provided as *offsets* to *edgeR* after transforming them to natural logarithms, allowing GC-content and copy-number biases to be incorporated directly into the differential accessibility model.

The design matrix included sample groups and TSS enrichment scores as a covariate. Tumor purity was modeled as a mixture of normal and cancer components, with the cancer component corresponding to the fraction of tumor cells and the remaining fraction representing BPH. Differential accessibility was tested using the quasi-likelihood framework implemented in *glmQLFit* and *glmQLFTest*. *p*-values were adjusted for multiple testing using the Benjamini–Hochberg method, with a significance threshold set at 0.1.

#### Analysis of mutation rates in chromatin compartments

Somatic mutation profiles across multiple cancer types were obtained from the PCAWG cohort^32^ and from a dataset of CRPC samples.^23^ Simple somatic mutation calls were downloaded from the ICGC data portal on 13 December 2023 (now discontinued, https://dcc.icgc.org/). The mutations were lifted from hg19 genome assembly to hg38 using the *rtracklayer* v1.66.0 R-package. To generate a spatial mutation profile for each dataset within the PCAWG metacohort, mutations were aggregated into 100kb genomic bins and total mutation counts per bin were computed.

#### Transcription factor binding data and enrichment analysis

Transcription factor binding data sets with at least 500 binding sites were obtained from the UniBind database,^52^ encompassing 4127 experiments across 253 TFs. ATAC-seq peaks were annotated by overlapping them with TF binding sites and genomic compartments. Overlaps were identified using the *findOverlaps* function from the *GenomicRanges* package.^81^ To define consensus binding sites for AR, FOXA1, HOXB13 and TP63 within our ATAC-seq peaks, we required that each peak overlap binding events from at least 25% of the UniBind experiments corresponding to the respective TF. Peaks satisfying this criterion were retained as consensus TF-bound regions. Enrichment significance was assessed using Fisher’s exact test. Similarly, chromHMM annotations generated by Gerhauser et al.,^51^ were used to annotate ATAC-peaks and tested for enrichment.

#### Block permutation analysis

To account for spatial dependence between neighboring genomic bins, selected compartment enrichment analyses were additionally assessed using a block permutation approach. Consecutive genomic bins sharing the same compartment state were identified as contiguous blocks and these blocks were randomly reordered. Importantly, the compartment annotations contained only genomic coordinates for which a compartment state was available; genomic regions with missing compartment assignments were therefore not explicitly permuted or imputed and were effectively excluded from the analysis. Conversely, gaps corresponding to missing regions remained excluded, and the reassignment of blocks could result in a block spanning positions separated by such gaps. We generated 10,000 randomized compartment configurations by independently shuffling the contiguous blocks within each chromosome and recalculated the log2 odds ratio for each permutation. Two-sided empirical *p*-values were calculated as the proportion of permutations with an absolute log2 odds ratio equal to or greater than that observed in the original data.

#### Analysis of H3Kme9 data

Published H3K9me3 data were obtained as processed bigWig files.^50^ BigWig tracks were lifted over from the hg19 to the hg38 genome build using the *rtracklayer v1.66.0* R package. Genome-wide signal was summarized in non-overlapping 100 kb bins by computing the total signal per bin, defined as the sum of the signal intensity for each record multiplied by its corresponding width in the bigWig file. To minimize copy number–driven effects, signal intensities were normalized within each chromosome arm by dividing by the arm-specific median. Finally, quantile normalization was applied across samples to ensure comparable signal distributions.

#### Loop detection

Loops were identified using Mustache,^54^ which detects locally enriched pixels from Hi-C contact maps. Mustache was run with the following parameters: *-r 10000*, *-pt 0.1*, *-st 0.8*, *-sz 1.6*, and *-d 2000000*. Loop calls across samples were merged using *pgltools* v2.2.0 merge with a 20 kb distance threshold.^71^ The unified loop coordinates were set to the median anchor positions. Loop strength was quantified per sample by summing raw contacts in a 3×3 pixel window centered on each loop (±10 kb at 10 kb resolution).

#### Differential loop analysis

Differential loops were detected by implementing copy number aware strategy described in diffHiC.^82^ Raw loop counts were imported into edgeR as a DGEList object.^68^ For each loop in each sample, we defined a loop-specific log2 ratio (loop-log2) by dividing the counts per million (CPM) for that loop by its mean CPM across all samples, followed by log2 transformation. For each loop anchor, coverage was derived from intrachromosomal contact matrices. Anchor-specific coverage log2 ratios (cov-log2) were computed analogously by normalizing each anchor’s coverage to its mean across samples and applying a log2 transformation.

A high-dimensional smooth surface was then fitted to predict loop-log2 values using the following predictors:

(i) the average cov-log2 across the two anchors,
(ii) the absolute difference in cov-log2 between anchors, and
(iii) the average loop CPM across all samples.

Model fitting was performed using the *locfit (locfit v1.5-9.10)* function with parameters span = 0.3, maxk = 500, and robust fitting (locfit.robust). The fitted values were transformed to natural logarithm and incorporated into the edgeR model as offsets, thereby jointly correcting for CNV-related bias and library size effects.

Following offset correction, dispersions were estimated and differential loop contacts were identified using quasi-likelihood testing (glmQLFit and glmQLFTest). *p*-values were adjusted for multiple testing using the Benjamini–Hochberg procedure, and differential loops were defined as those with an adjusted *p*-value < 0.05.

#### Structural variation detection from Hi-C data and neoloop and outlier gene expression detection

Structural variation detection from Hi-C data was performed using Breakfinder (https://github.com/dixonlab/hic_breakfinder) to identify genomic breakpoints.^28^ For samples with replicates, breakpoints from the replicate with higher sequencing depth were used for further analysis. Assembly of breakpoint-associated regions and neoloop detection were carried out using NeoLoopFinder (https://github.com/XiaoTaoWang/NeoLoopFinder).^27^ Genes were annotated as neoloop-associated if they overlapped at least one anchor of a detected neoloop; all loops in the assembled regions were considered for annotation, including those not directly spanning the breakpoint. Outlier gene expression was defined on a per-sample basis using Tukey’s rule, whereby genes lying more than 1.5× the interquartile range (IQR) above the third quartile for that gene across the cohort were classified as over-expressed outliers.

#### Association of interchromosomal translocations with compartments

Structural variant calls for CRPC were obtained from a previously published cohort.^23^ Interchromosomal translocations were extracted and breakpoint genomic coordinates were assigned to A/B-compartment labels using Hi-C eigenvector tracks at 100kb resolution. For each translocation, the compartment pair was defined as AA, BB, or AB (mixed), based on the compartment identities of the breakpoint pairs. To test whether interchromosomal translocations occurred non-randomly with respect to compartment identity, we generated an expected distribution by shuffling compartment labels of the 100kb bins within each chromosome. Expected frequencies for each compartment pair and empirical *p*-values were derived from 1000 shuffles.

To examine whether translocation-linked loci are spatially proximal prior to rearrangement, we quantified interchromosomal contact frequencies using KR-normalized interchromosomal Hi-C maps averaged across the BPH samples. For each observed translocation, the normalized Hi-C contact value between the two breakpoint bins was recorded in 1 Mb resolution. Compartment-specific comparisons were performed by stratifying translocations into AA, BB, and AB groups. To establish a random background, breakpoint coordinates were randomly re-paired to generate pseudo-translocations. Contact enrichments were calculated by comparing observed and random background distributions using the Wilcoxon signed-rank test.

### Quantification and statistical analysis

Statistical analyses and data visualizations were performed in R (4.4.1) unless otherwise specified. Used software and package versions are specified in the relevant method details subsections. Differential analyses were performed using the statistical models and tests described for each analysis. For comparisons of frequencies or proportions, Fisher’s exact test was used. For comparisons of continuous or ordinal values, the Wilcoxon rank-sum test was used. Where applicable, *p*-values were adjusted for multiple testing using the Benjamini–Hochberg procedure. Statistical significance thresholds and number of subjects are reported in the corresponding method details subsections and figure legends. Permutation-based analyses used empirical *p* values calculated as described in the STAR Methods subsections.

## Supplemental information

Document S1. Figures S1–S22

Table S1. Sample information and estimated tumor purities, related to Figure 1

Table S2. Hi-C sequencing statistics, related to Figure 1

Table S3. Hi-C sample concordance, related to Figure 1 Sample concordance is calculated between all samples and replicates from chromosome 1 with HiCRep.

Table S4. A/B compartment scores across sample, related to Figure 1 First principal components of copy-number-segment-corrected Hi-C matrixes in 100 kb resolution.

Table S5. Blacklisted regions excluded from the analysis, related to Figure 1

Table S6. Structural variant breakpoints detected by breakfinder, related to Figure 1

Table S7. Compartment annotations, related to Figure 2 Annotated compartment class, adjusted *p* values from differential compartment analysis, and mean compartment scores in BPH and at an estimated 100% tumor purity.

Table S8. Leave-one-sample-out analysis of differential compartments, related to Figure 2 Differential compartment analysis was repeated with each sample excluded, and the results were compared with those obtained using all samples.

Table S9. Prostate cancer up-regulated genes located in activating B compartments, related to Figure 3

Table S10. Enrichment of transcription factor binding sites in activating B compartments, related to Figure 4 Transcription factor binding data obtained from the UniBind database.

Table S11. Effect of transcription factor expression on compartment shifts, related to Figure 4 Linear regression models quantifying the additional variance in activating compartment shifts explained by transcription factor expression beyond tumor purity alone.

Table S12. Unified set of loops detected across samples, related to Figure 4
