# Supplementary material for: Chromatin compartment shifting is recurrent and associated with epigenetic reprogramming in prostate cancer
Source: iScience. 2026 Nov 6;29(11):118155. doi: 10.1016/j.isci.2026.118155 (PMC13761249; doi:10.1016/j.isci.2026.118155)
Supplement: Supplementary file 1 [file mmc1.pdf]

iScience, Volume *29*

## Supplemental information

# Chromatin compartment shifting is recurrent and associated with epigenetic reprogramming in prostate cancer

**Tuomo Virtanen, Ebrahim Afyounian, Riikka Nurminen, Joonas Uusi-Mäkelä, Antti Kiviaho, Joonatan Sipola, Francesco Tabaro, Juha Kesseli, Teuvo Tammela, Teemu J. Murtola, Alfonso Urbanucci, Tapio Visakorpi, Kirsi Rautajoki, and Matti Nykter**

# Supplementary figures

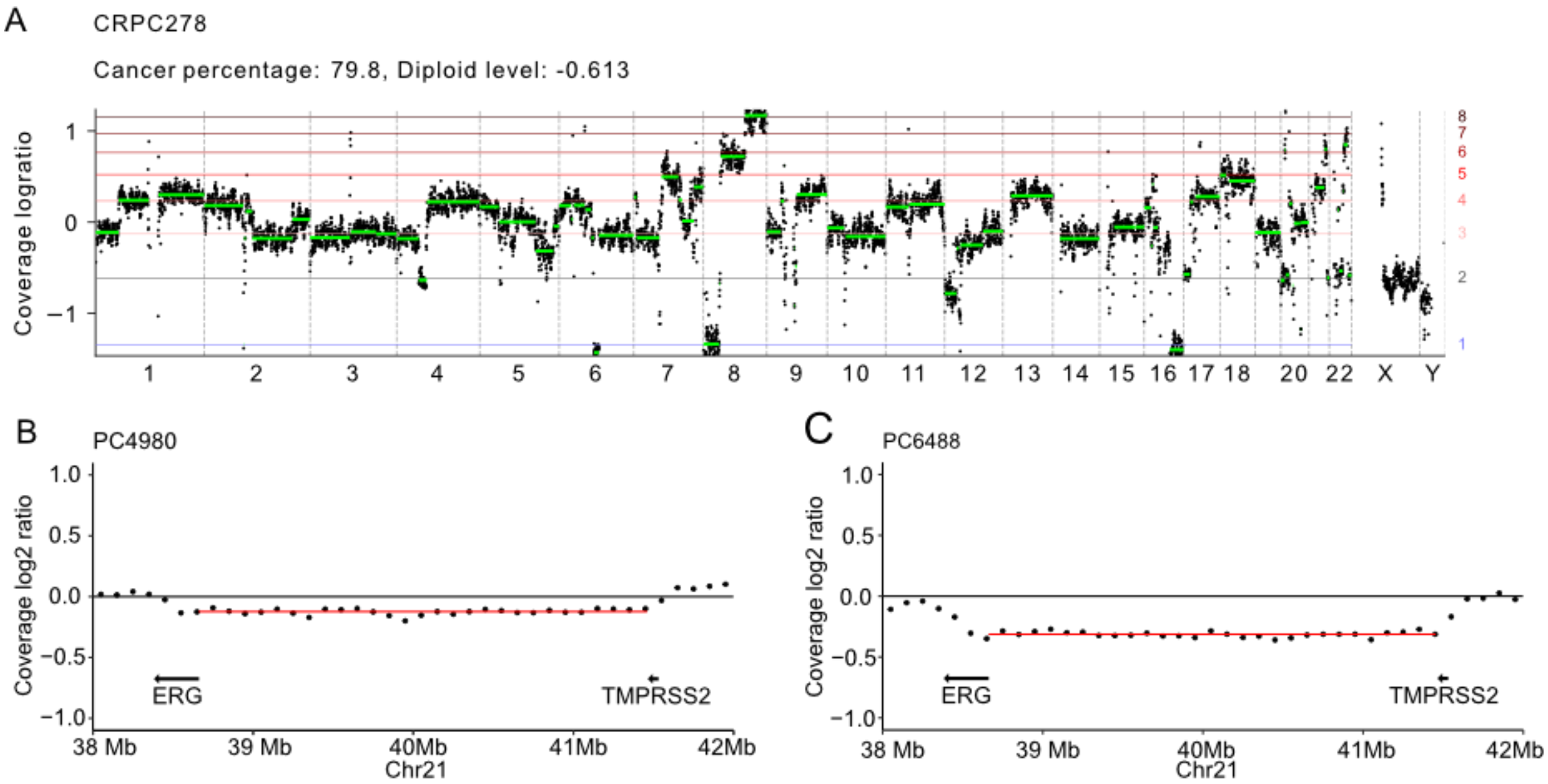


**Figure S1. Tumor purity estimation from Hi-C coverage profiles, related to Figure 1.**

(**a**) Example of a Hi-C sequencing sample with high tumor content. Horizontal lines indicate the estimated copy numbers, given the inferred tumor purity and diploid level. (**b–c**) Two samples exhibited minimal copy number variation, making copy number modeling difficult. Both samples carried a *TMPRSS2–ERG* fusion with an associated visible deletion. In these cases, tumor purity was estimated from the deletion under the assumption of a monoallelic event in a diploid genome.

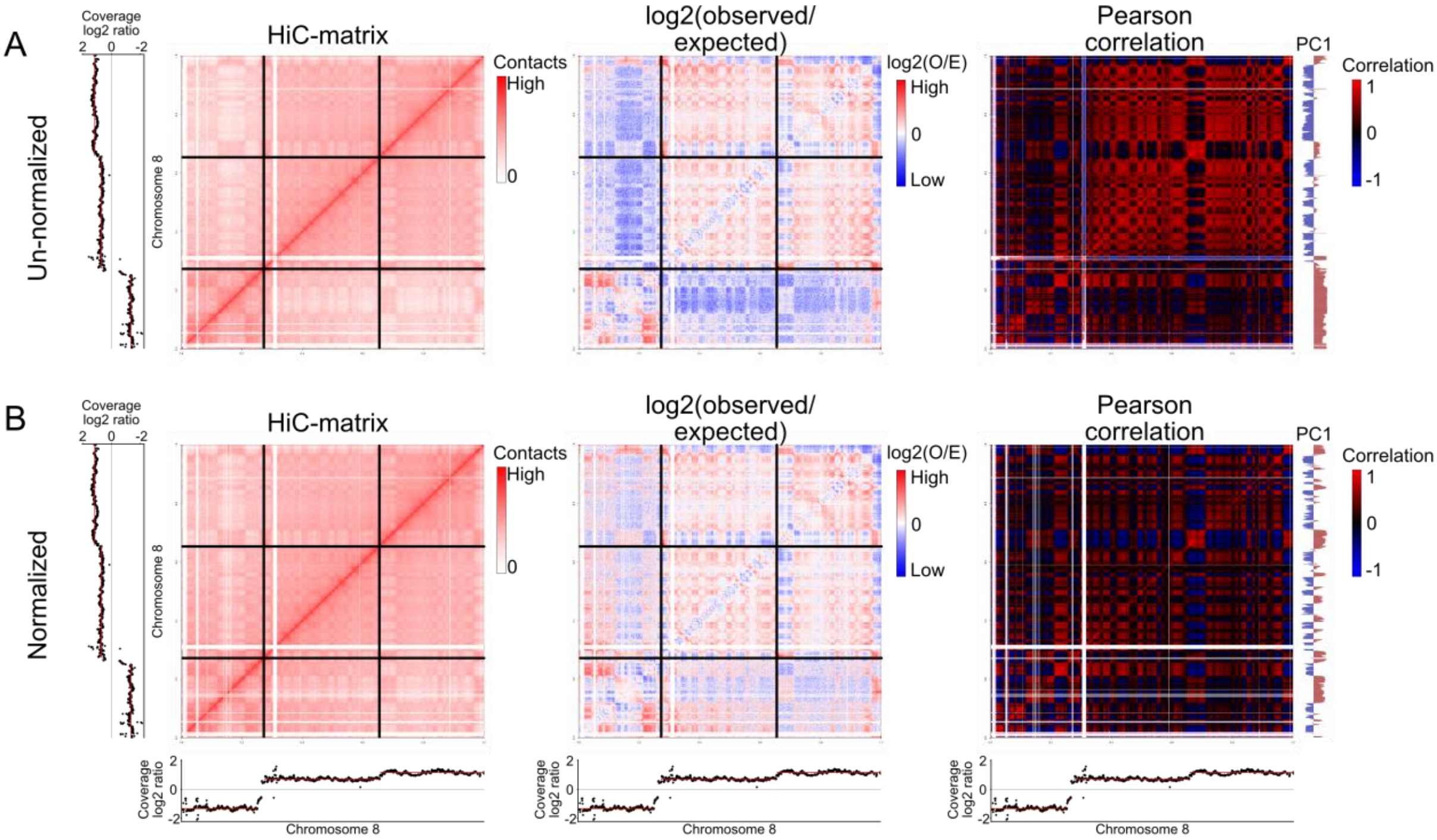


**Figure S2. Copy number–aware normalization of Hi-C contact matrices, related to Figure 1.**

(**a**) Example of an unnormalized Hi-C contact matrix and the corresponding compartment profile. (**b**) Hi-C matrices after normalization by copy number segments. The matrix is divided into a grid defined by copy number segments, and for each region in the grid, the average observed/expected contact value is used as a normalization factor. After normalization, the estimated compartments more closely resemble the visible plaid pattern in the Hi-C matrix.

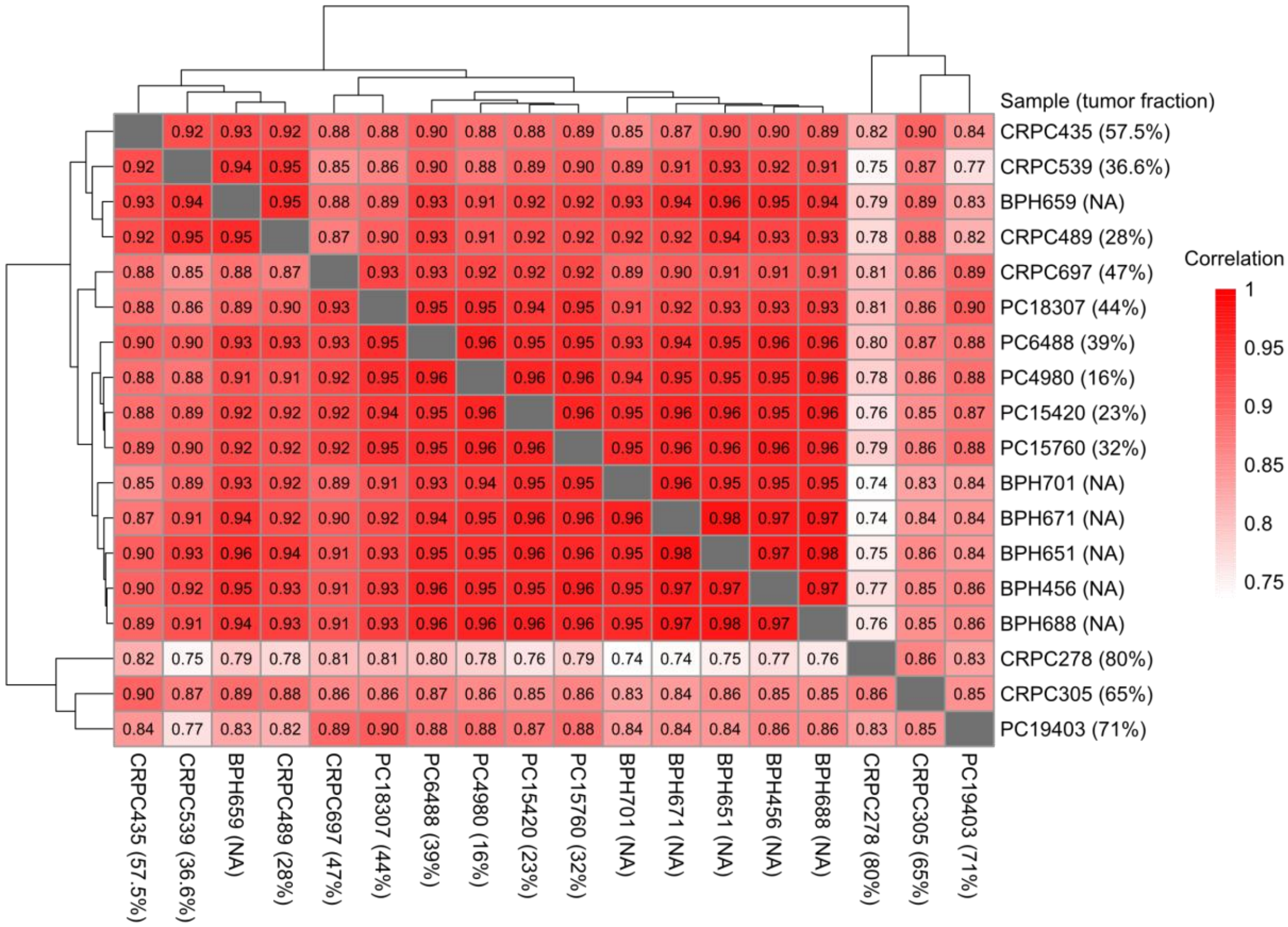


**Figure S3. A/B-compartments are highly correlated between samples, related to Figure 1**.

Pearson correlation matrix of the AB-compartment scores. Rows and columns of the matrix are sorted by hierarchical clustering using correlation as a distance metric. Three samples with the highest tumor purity cluster separately from other samples.

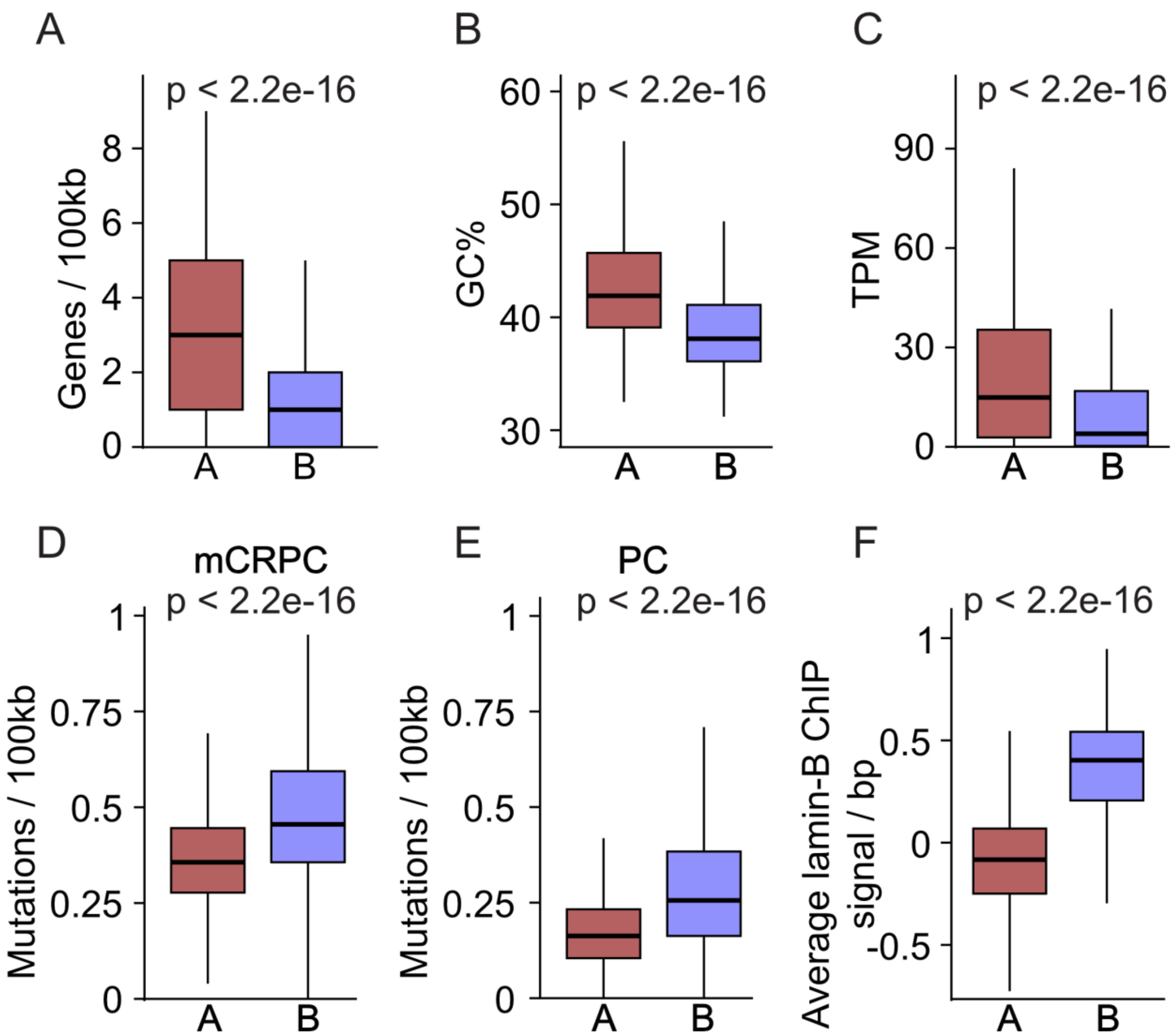


**Figure S4. A/B-compartments correlate with known biology, related to Figure 1.**

(**a**) Number of genes in 100kb bins belonging to A- and B-compartments. (**b**) GC fraction in 100kb bins belonging to A- and B-compartments. (**c**) Transcripts per million (TPM) in genes located in A- or B-compartments. (**d**) Average number of mutations in 100kb bins from patients with metastatic castration resistant prostate cancer (mCRPC). [1] (**e**) Average number of mutations in 100kb bins from patients with primary prostate cancer (PC). [2] (**f**) Average lamin-B chip signal per bp in 100kb bins belonging to either A- or B-compartments.

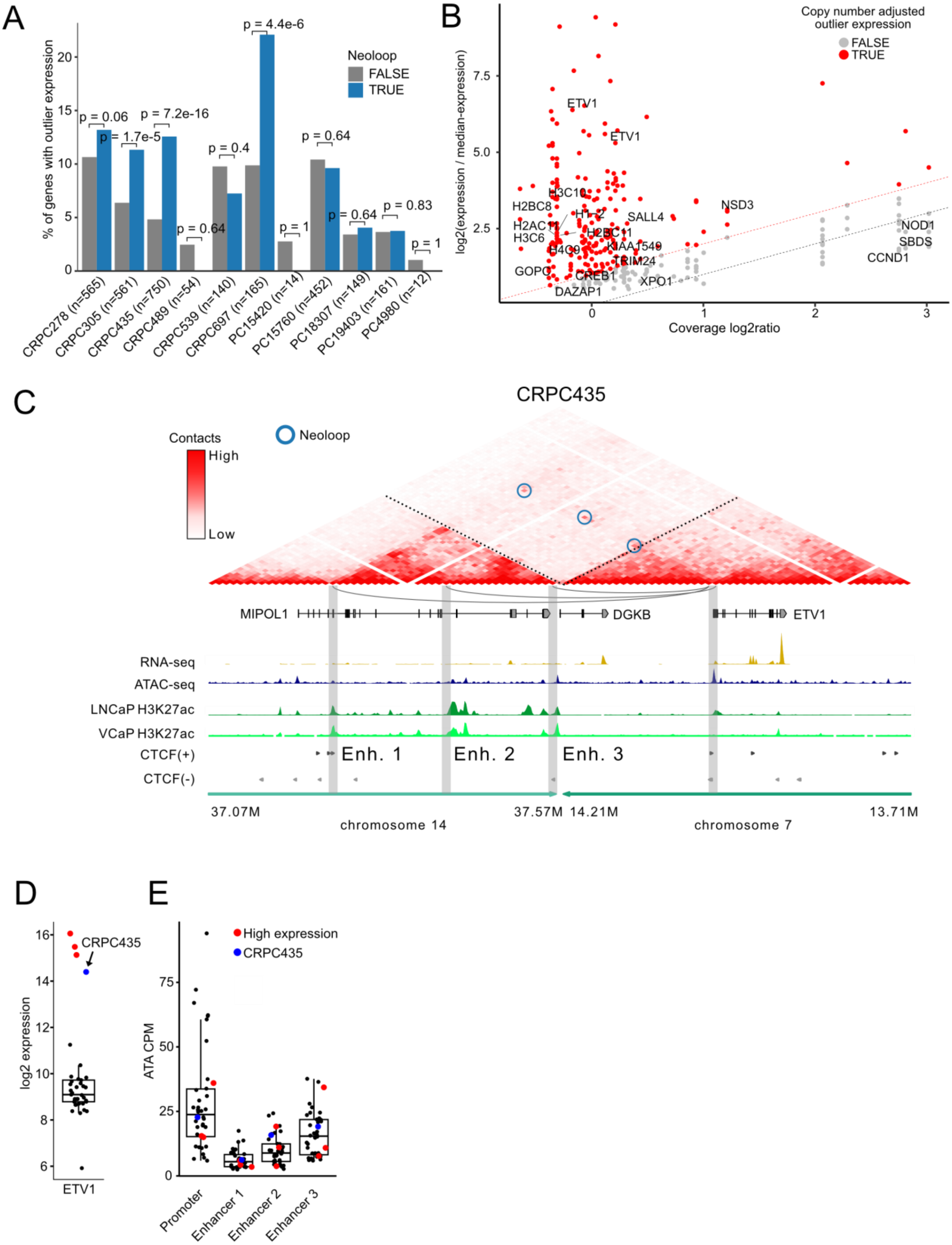


**Figure S5. Enhancer hijacking drives oncogene expression in castration resistant prostate cancer, related to Figure 1.**

(**a**) Genes with outlier expression are enriched at neoloops formed near structural variants. Bar plot shows the percentage of genes with outlier expression among genes overlapping neoloops compared with all other genes. *P*-values were calculated using Fisher's exact test. The number of genes associated with neoloops are shown within the brackets. (**b**) Most

outlier expression events at neoloops cannot be explained by copy-number variation. Scatter plot showing each gene's coverage log ratio (x-axis) versus its log2 fold change relative to the cohort median (y-axis). The gray line represents the expected linear relationship (slope = 1). The red line marks the threshold where observed expression is at least twice what would be predicted from copy-number changes alone. Genes exceeding this threshold are highlighted in red. Genes annotated as oncogenes are labeled in the plot. (**c**) ETV1 is activated by enhancer hijacking. Hi-C contact map reassembled around the translocation event involving the *ETV1* locus and the *MIPOL1* gene. The *ETV1* promoter forms interactions ("neoloops") with three distal enhancers originating from chromosome 14, indicated by circles and connecting arcs. Shown below are gene expression and ATAC-seq tracks, along with H3K27ac ChIP-seq profiles from LNCaP (GEO sample GSM4087689) and VCaP (GEO sample GSM4462721) cells [3],[4]. LNCaP cells have previously been reported to harbor a similar enhancer-hijacking event [5] . Consistent with this, histone H3 lysine 27 acetylation (H3K27ac) signal is elevated at the *ETV1* promoter in LNCaP compared to the *ETV1*-negative cell line VCaP. (**d**) Gene expression of ETV1 across the RNA-cohort. Samples with elevated expression are highlighted with red and blue. Blue color indicates the sample with ETV1-MIPOL translocation. (**e**) Chromatin accessibility at the *ETV1* promoter and distal enhancers. ATAC-seq chromatin accessibility (CPM = counts per million) is shown for the *ETV1* promoter and the three enhancers highlighted in panel (c). Samples with elevated *ETV1* expression are marked in red and blue, with blue indicating the sample carrying the *ETV1–MIPOL1* translocation. High *ETV1* expression is not accompanied by increased accessibility at the *ETV1* promoter, and the *ETV1–MIPOL1* fusion does not alter accessibility at the enhancers located within the *MIPOL1* locus.

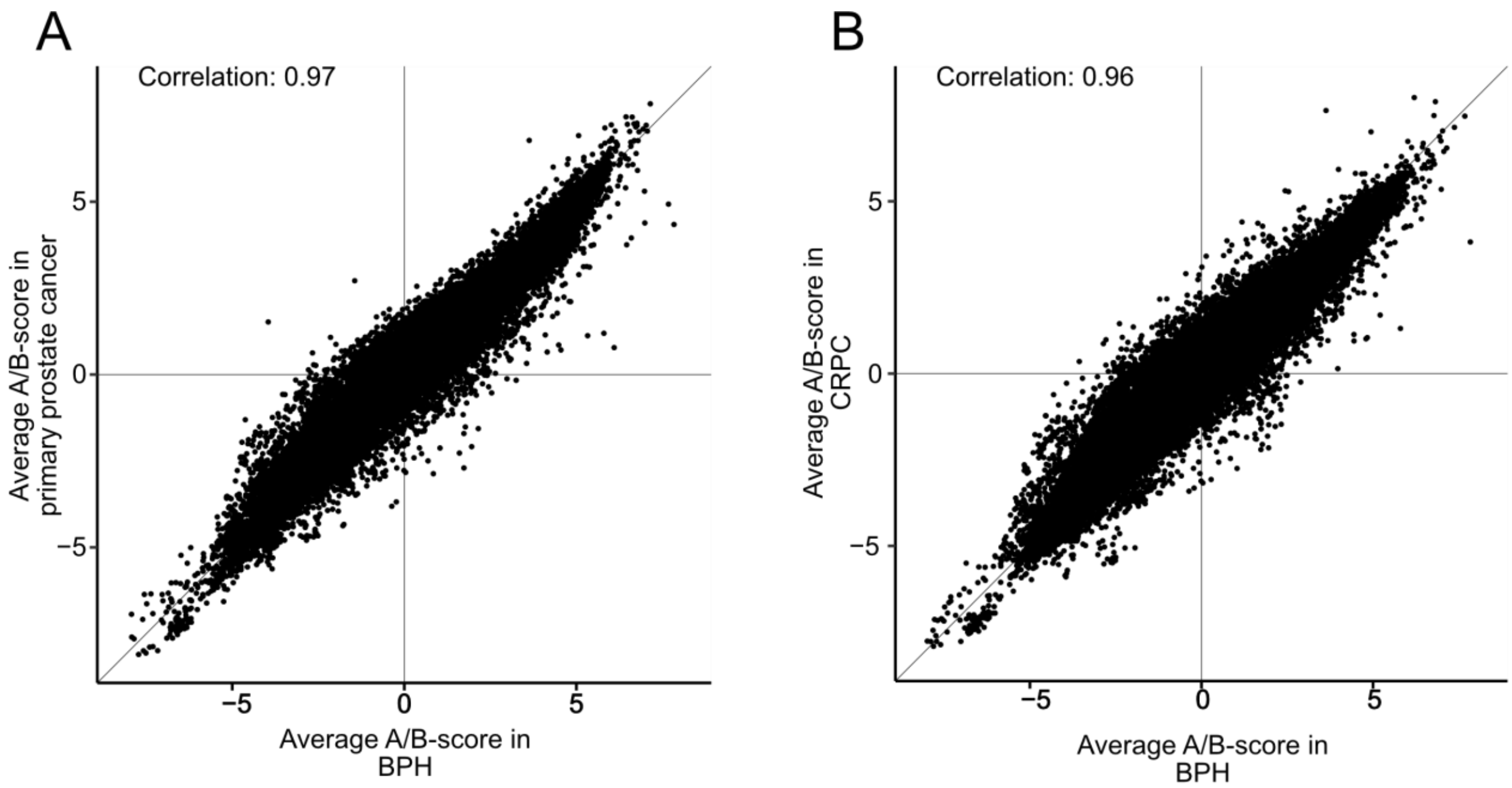


**Figure S6. A/B-compartments are highly correlated between benign prostatic hyperplasia (BPH) and prostate cancer, related to Figure 2.**

(**a**) The average A/B score (first principal component of the Pearson correlation matrix) in BPH vs primary prostate cancer. (**b**) The average A/B-score in BPH vs castration resistant prostate cancer (CRPC).

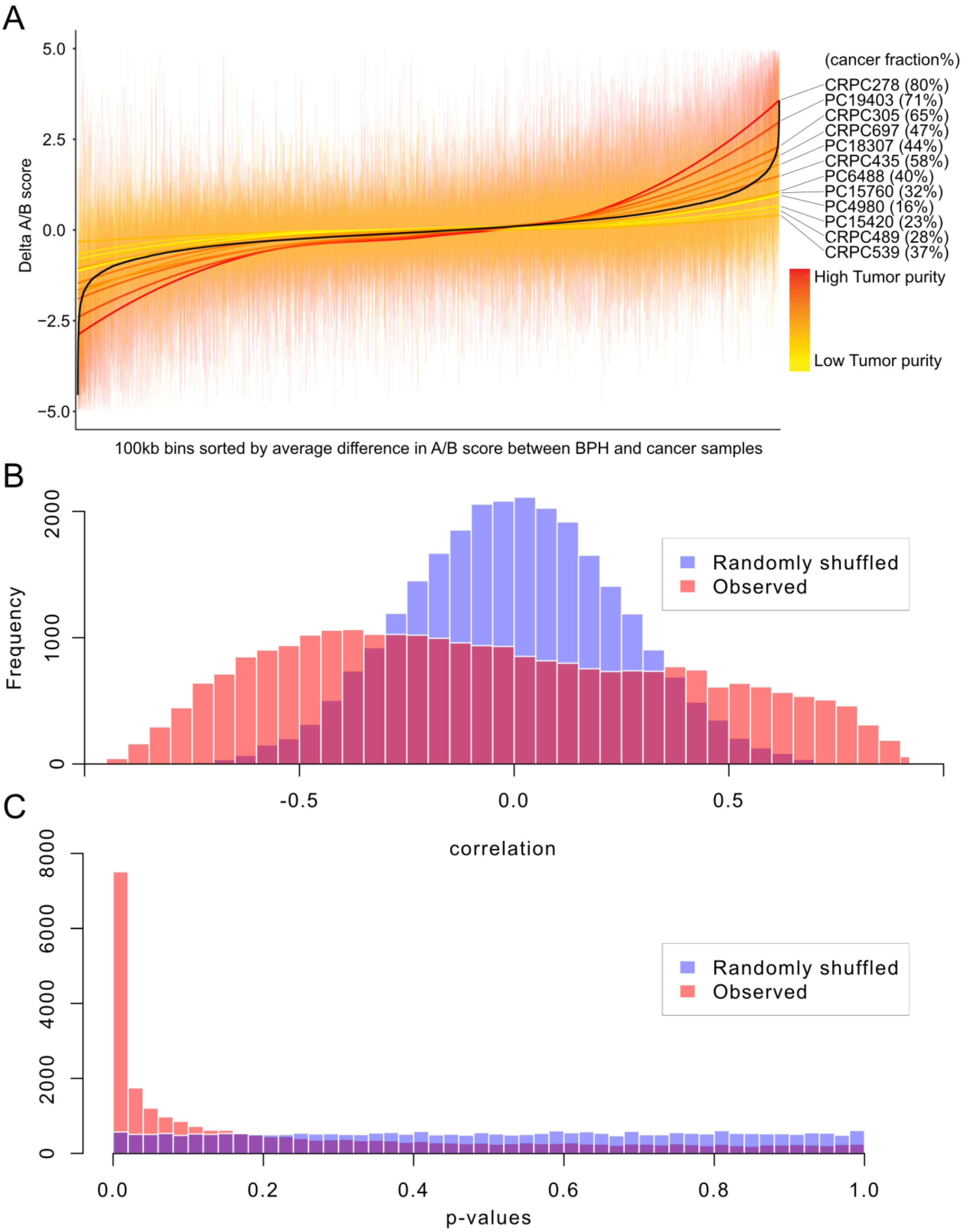


**Figure S7. Compartmentalization differences correlate with tumor purity, related to Figure 2.**

(**a**)100kb bins are ordered by the difference in average A/B signal between BPH and all cancer samples combined. The black line shows the average difference, while transparent lines represent individual cancer samples relative to the BPH average. Solid colored lines indicate locally smoothed trends (local regression, *locfit* package in R). Sample identities and their corresponding tumor purities are shown on the right. (**b**) Histograms showing the

distribution of Pearson correlations between PC1 values and tumor purity across genomic bins for the observed and randomly shuffled tumor purity labels. (**c**) Histograms showing the corresponding distribution of correlation *p*-values. Observed tumor purity labels yield substantially stronger correlations and more significant *p*-values than randomly shuffled tumor purity labels, indicating a non-random association between PC1 and tumor purity.

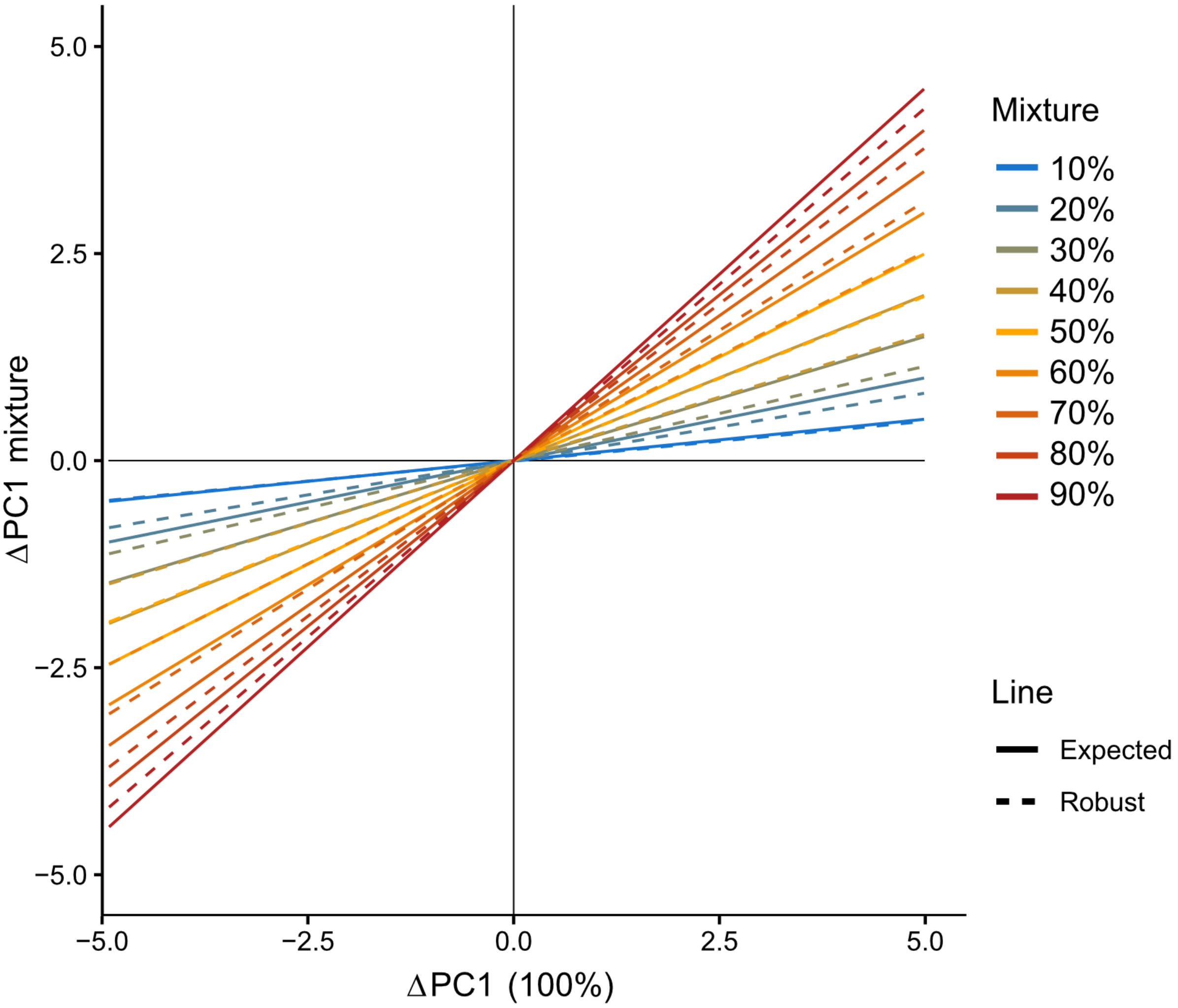


**Figure S8. Validation of PC1 compartment-score dilution using synthetic Hi-C mixtures, related to Figure 2.**

Synthetic Hi-C datasets were generated by randomly subsampling Hi-C contacts from BPH456 and CRPC278 (a high-tumor-purity tumor sample), and combining the two datasets at defined CRPC fractions of 10–100% while maintaining a total of 500 million contacts per mixture. A/B-compartment PC1 (first principal component) scores were calculated for each synthetic mixture using the same analysis pipeline as for the primary Hi-C datasets. For each genomic bin, PC1 values were expressed relative to the BPH456 reference (ΔPC1). The x-axis represents the ΔPC1 observed in the 100% CRPC278 dataset, and the y-axis represents the corresponding ΔPC1 in each synthetic mixture. Each data point (not shown) represents a 100kb genomic bin retained after removal of bins with missing values and sex chromosomes. Solid lines show robust linear-regression fits, and dashed lines indicate the theoretical expectation for linear dilution of the CRPC-associated compartment signal, with slopes corresponding to the simulated CRPC fraction. The observed relationships closely followed the expected dilution lines across mixture proportions, with modest attenuation of the observed slopes relative to the theoretical values. Correlations were strongest at higher tumor fractions and decreased at lower fractions, consistent with increased sampling noise in lower-depth effective tumor components. This analysis supports the linearity assumption between Hi-C PC1 values and tumor purity.

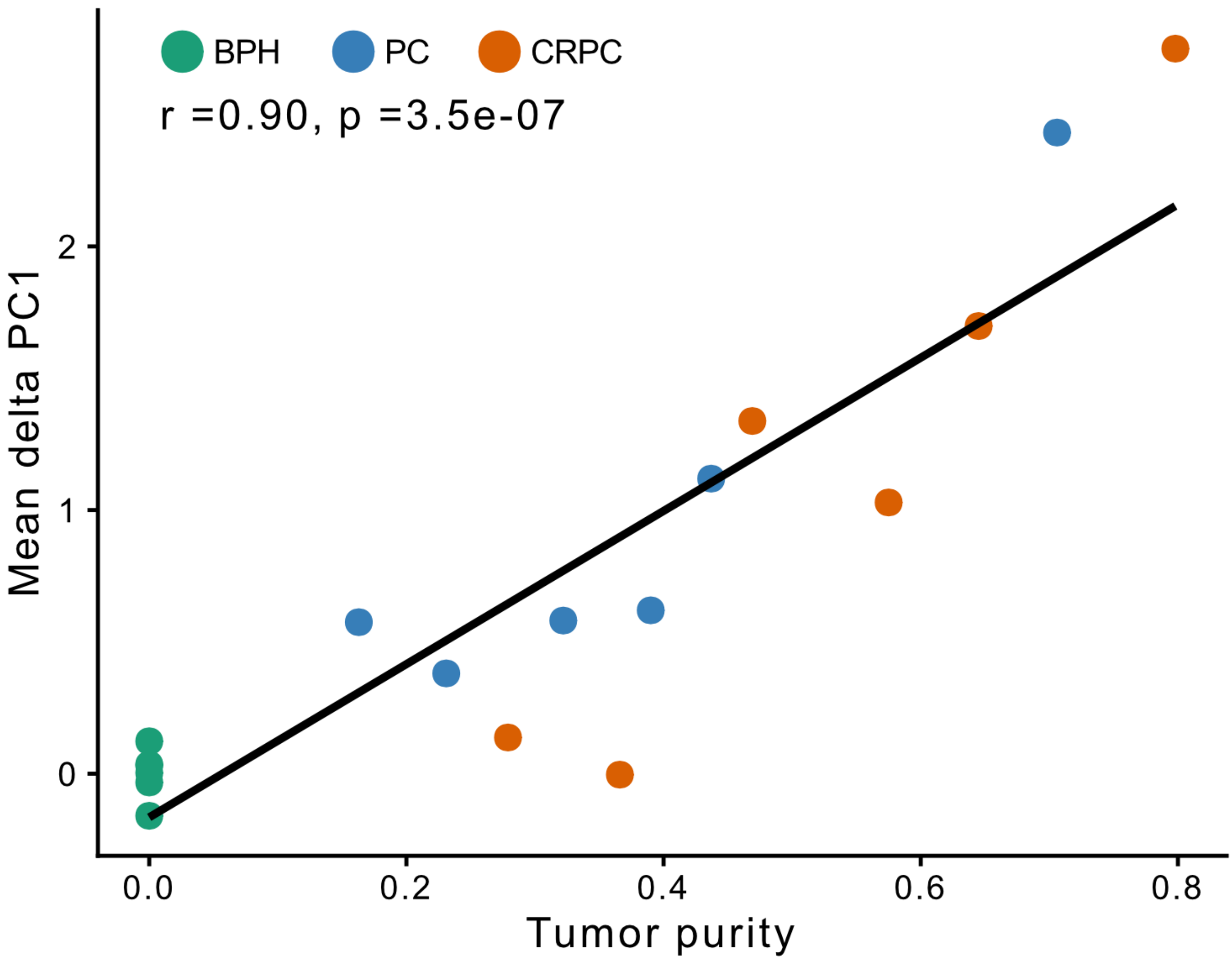


**Figure S9. Changes in the first principal component (PC1) in activating B-compartments show a linear relationship with tumor purity, related to Figure 2.**

The plot shows the relationship between the average change in PC1 per sample and tumor purity, with PC1 changes calculated relative to the average PC1 values of BPH samples. Correlation (r) and the p-value are shown in the figure. BPH=benign prostatic hyperplasia, PC=primary prostate cancer, CRPC=castration resistant prostate cancer.

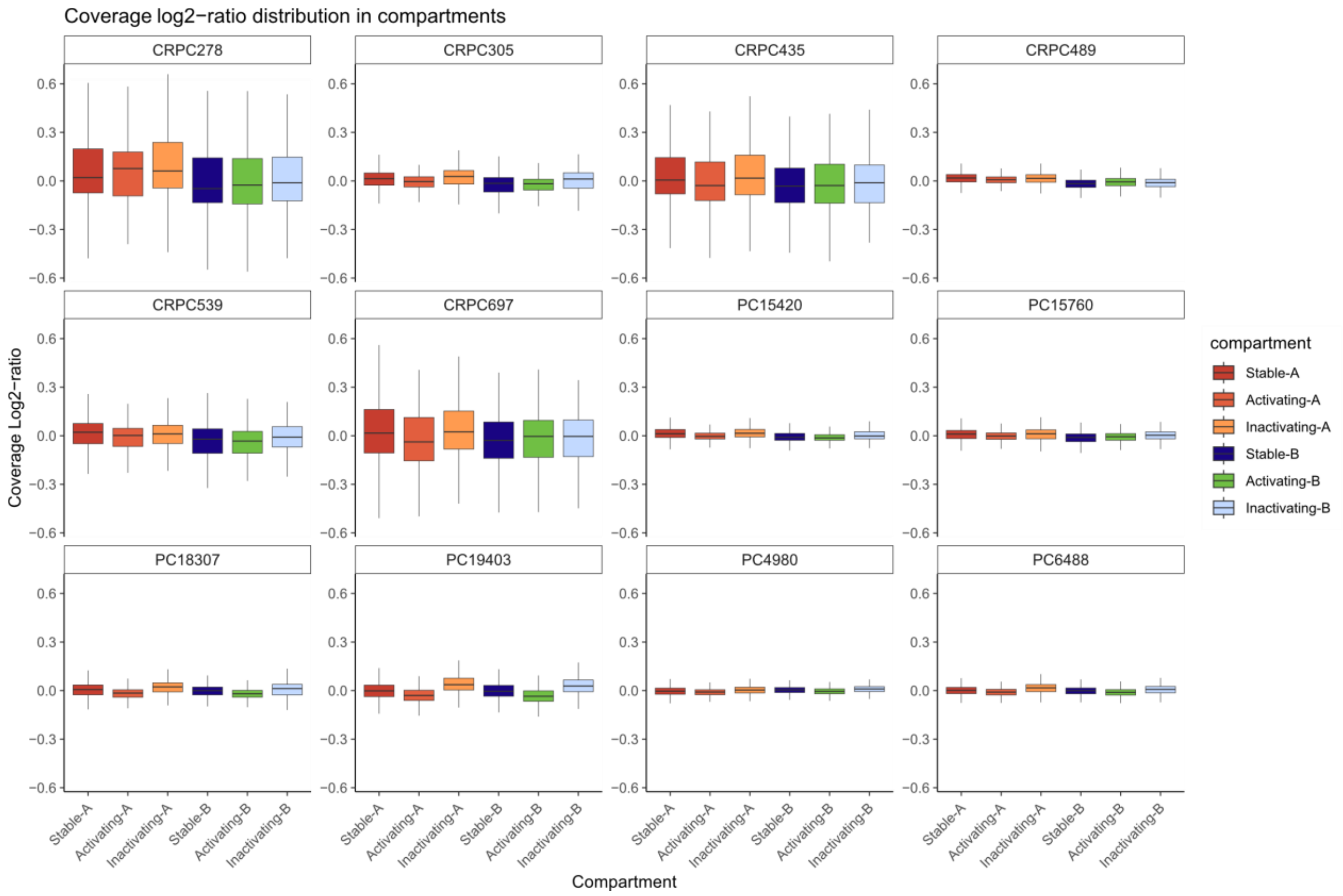


**Figure S10. Activating B-compartments are not associated with copy number gains, related to Figure 2.**

Box plots of coverage log2 ratios of 100kb bins grouped by their annotated compartment across all cancer samples.

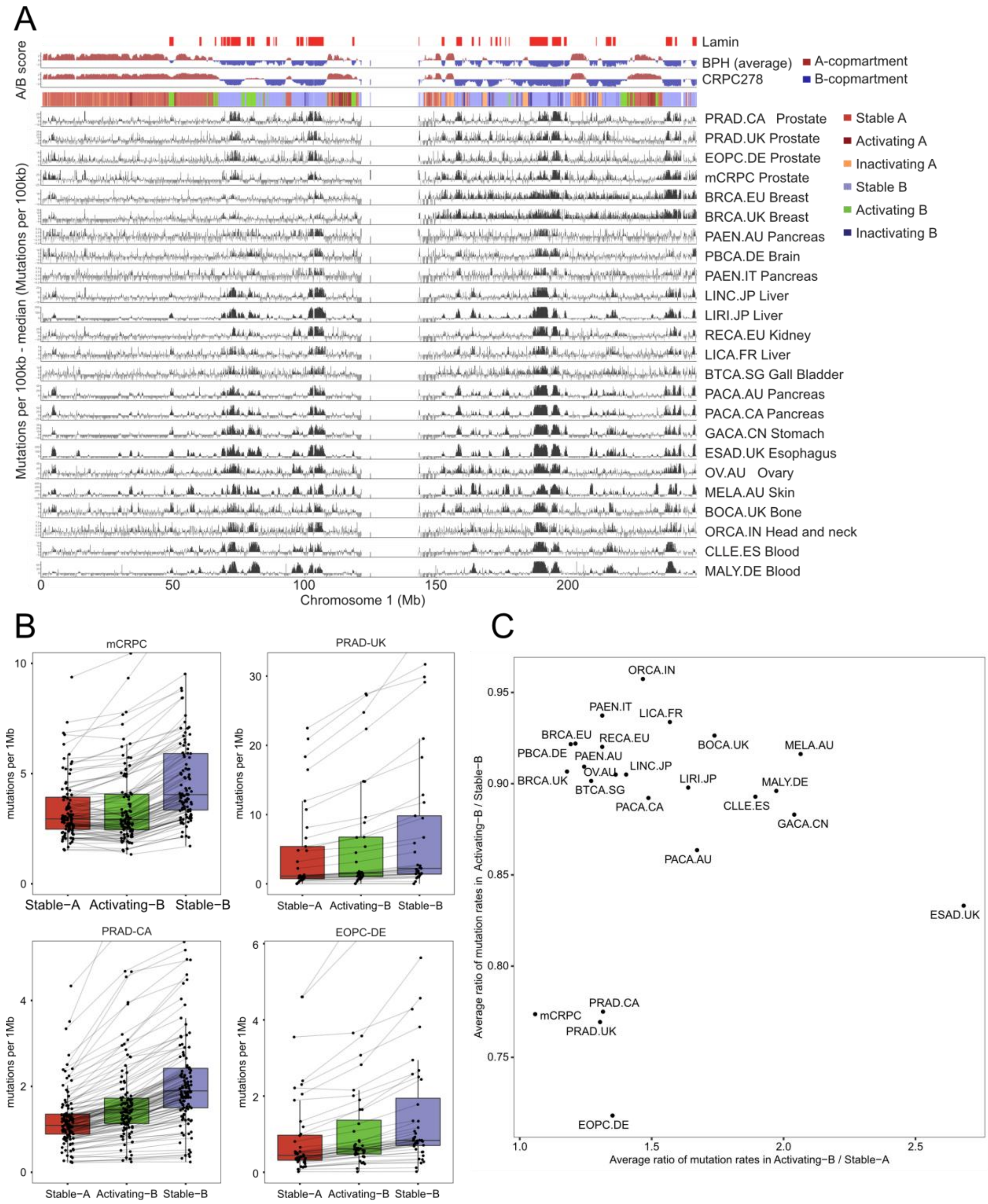


**Figure S11. Mutation rates in prostate cancer correlate with compartment status, related to Figure 2.**

(**a**) Overview of mutation rates across the PCAWG dataset and a published mCRPC dataset [1]. Black and grey bar plots show the total number of mutations per 100 kb bin detected in each dataset. For visualization, mutation rates were normalized by subtracting the median mutation rate across all 100kb bins. At the top of the panel, lamin consensus sites, the average A/B-compartment score in benign prostatic hyperplasia samples, and the A/B score of the CRPC sample with the highest tumor purity are shown. Compartment categories are

indicated by colored segments. (**b**) Mutation rates of different compartments across four prostate cancer datasets. Each point represents an individual sample, and matching samples are connected by lines. (**c**) Prostate cancer exhibits decreased mutation rates in activating B-compartments relative to stable B-compartments. For each sample, the ratio of mutation rates between activating B- and stable B-compartments, as well as between activating B- and stable A-compartments, was calculated. The average values for each dataset are displayed in the scatter plot.

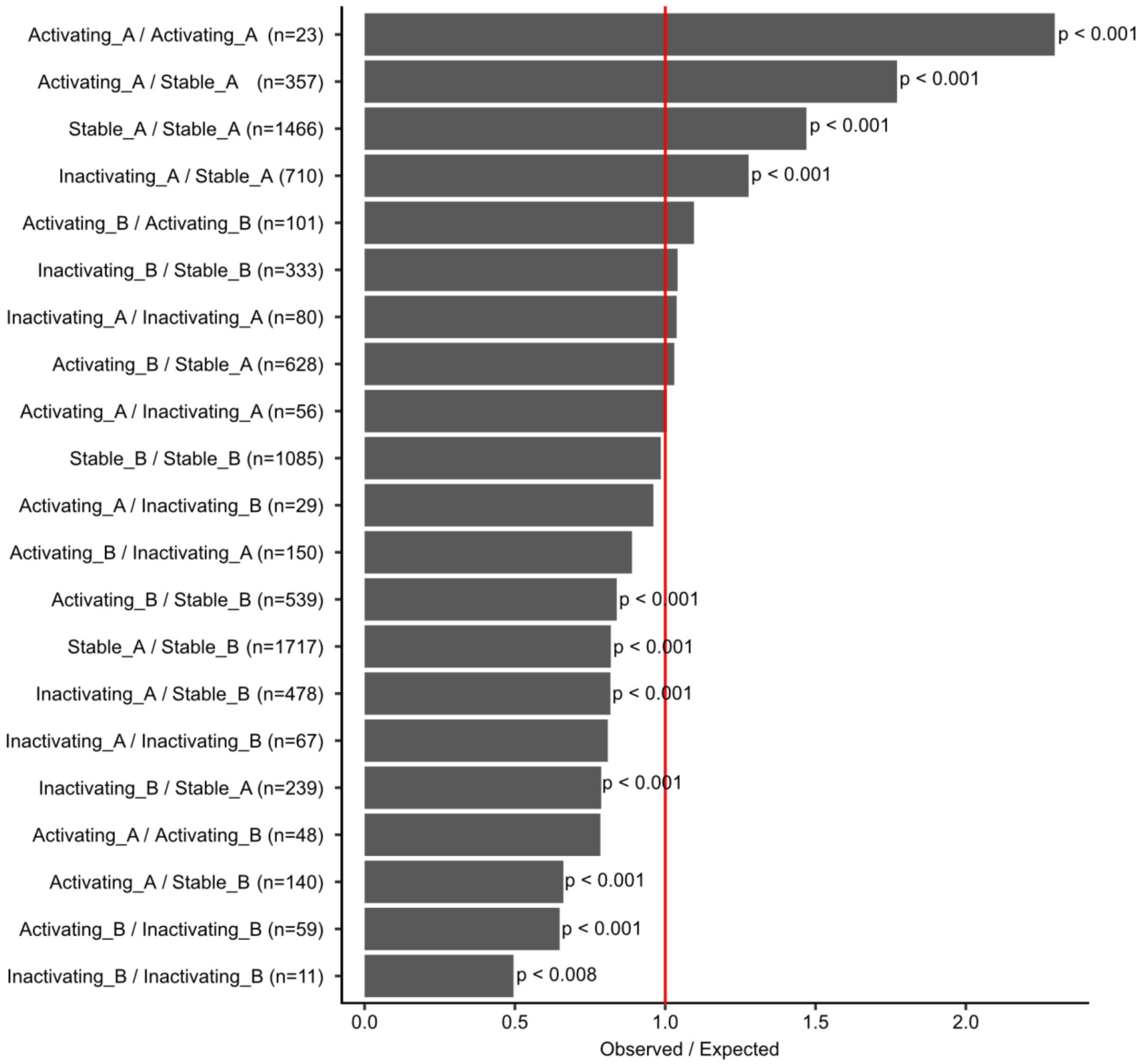


**Figure S12. Enrichment of interchromosomal translocations between compartment classes, related to Figure 2.**

Observed interchromosomal translocation breakpoint pairs from published dataset were classified according to the compartment state at each breakpoint [1]. We generated 1,000 randomized datasets by randomizing the compartment assignments of translocation pairs within their corresponding chromosomes while preserving the chromosome-specific compartment composition, and compared the observed number of translocations for each compartment-state pair with the randomized expectation. The observed/expected ratio indicates enrichment (>1) or depletion (<1). Translocations between Activating B and A compartments showed no significant enrichment or depletion, whereas translocations between Stable A and B compartments were significantly depleted. These patterns are consistent with the repositioning of Activating B compartments toward A compartments. Significant P-values ($p < 0.01$) are shown and were calculated from the 1,000 randomizations using one-sided tests for enrichment or depletion, as indicated in the figure.

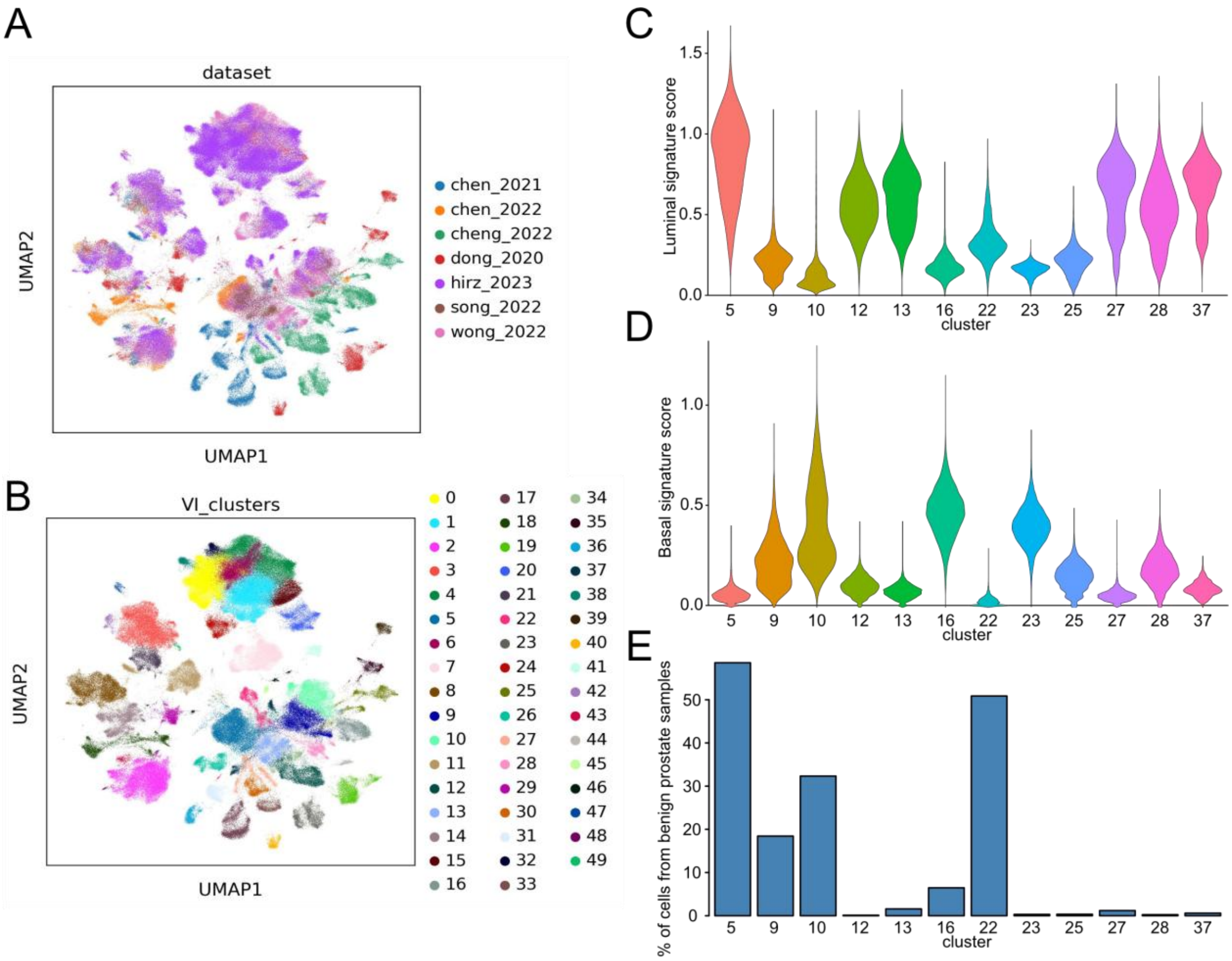


**Figure S13. Metacohort of prostate cancer single-cell RNA-seq data, related to Figure 3.**

Data previously analyzed by Kiviaho et al. [6] . (**a–b**) UMAP(Uniform Manifold Approximation and Projection for Dimension Reduction) representation of the integrated dataset. Each point corresponds to a single cell and is colored by dataset of origin (**a**) or by predicted cluster identity (**b**). (**c**) Violin plot showing luminal signature scores in clusters annotated as epithelial cells. (**d**) Violin plot showing basal signature scores in clusters annotated as epithelial cells. (**e**) Bar plot depicting the percentage of cells derived from benign samples.

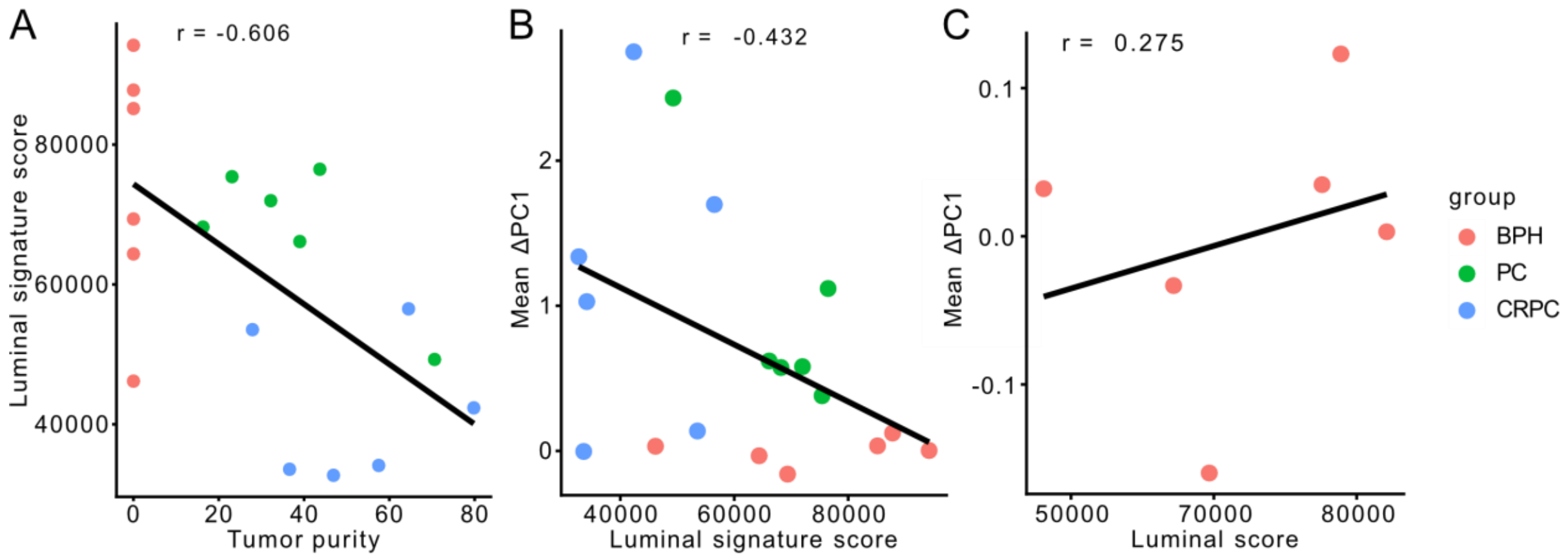


**Figure S14. Relationship between luminal gene expression signature and compartment shifting, related to Figure 3.**

(**a**) Scatter plot showing the relationship between luminal score and tumor purity in benign prostatic hyperplasia (BPH), primary prostate cancer (PC), and castration resistant prostate cancer (CRPC) samples. BPH samples exhibited the greatest variability in luminal scores. Overall, luminal score was negatively correlated with tumor purity, with CRPC samples showing the lowest luminal scores. This may reflect transcriptomic reprogramming and attenuation of the androgen receptor (AR)-associated luminal expression program during CRPC progression. The luminal signature comprises a gene set associated with normal luminal prostate cells, as defined by Kiviaho et al. [6] . (**b–c**) Relationship between luminal score and the mean deviation in the first principal component (PC1) of activating B compartments, shown for all samples (**b**) and BPH samples alone (**c**). No clear association was observed between luminal score and deviations from the average activating B-compartment signal in BPH samples. However, the statistical power is limited.

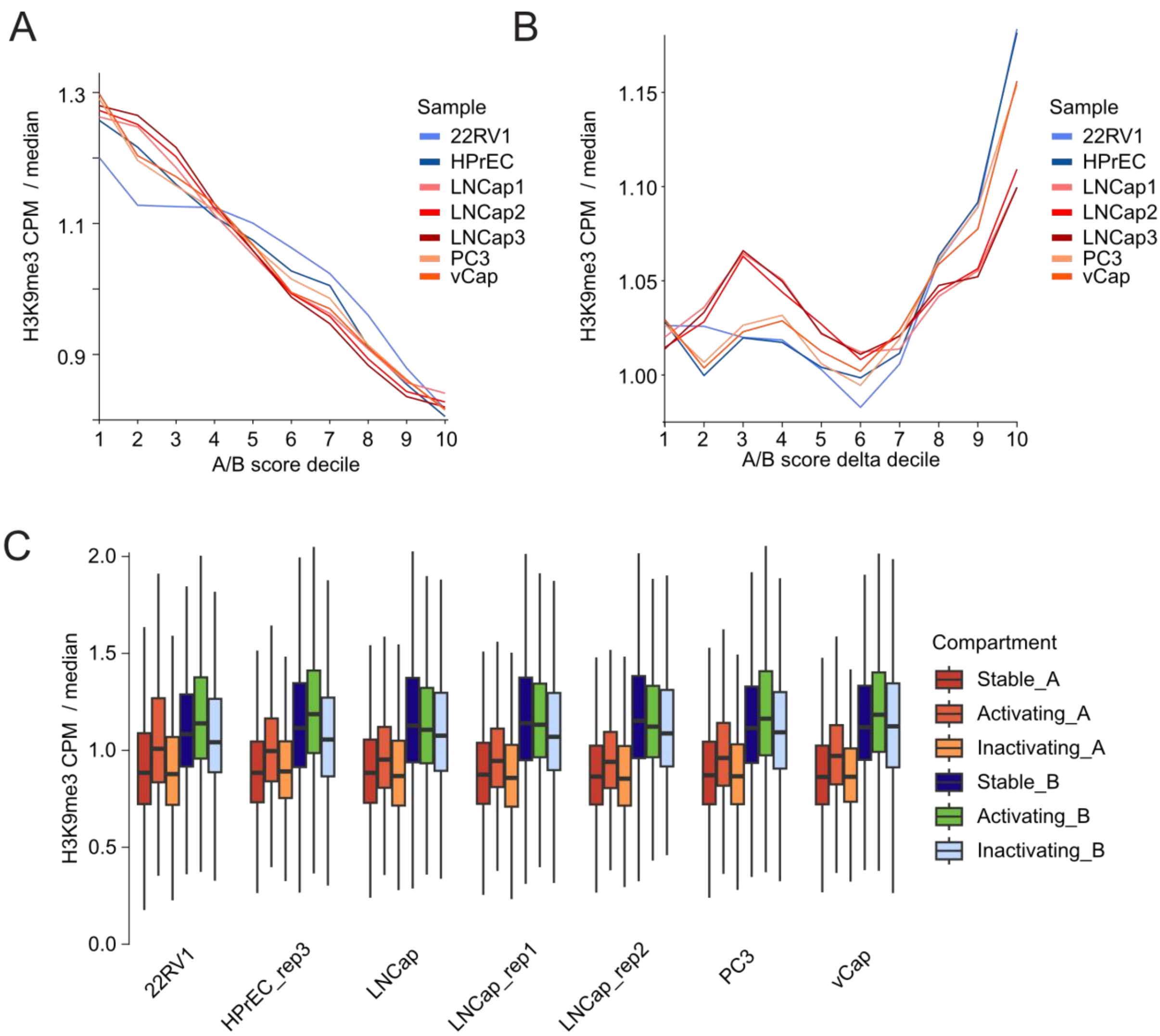


**Figure S15. Association of H3K9me3 with compartment shifts, related to Figure 4.**

(**a**) Trimethylation of lysine 9 on histone H3 (H3K9me3) shows a strong correlation with A/B-compartment score. The y-axis represents the mean H3K9me3 signal across 100kb genomic bins [7] , stratified by A/B score deciles. (**b**) Compartments that become activated in LNCaP samples exhibit reduced H3K9me3 levels. The y-axis shows the mean H3K9me3 signal in 100kb bins stratified by deciles of the A/B score change, where higher values indicate increased A/B scores in cancer relative to normal samples. Notably, these activating compartments display the highest H3K9me3 levels in normal cell lines, consistent with epigenetic repression of lamin-associated regions. (**c**) Barplot of H3K9me3 levels in 100kb bins annotated to different compartments.

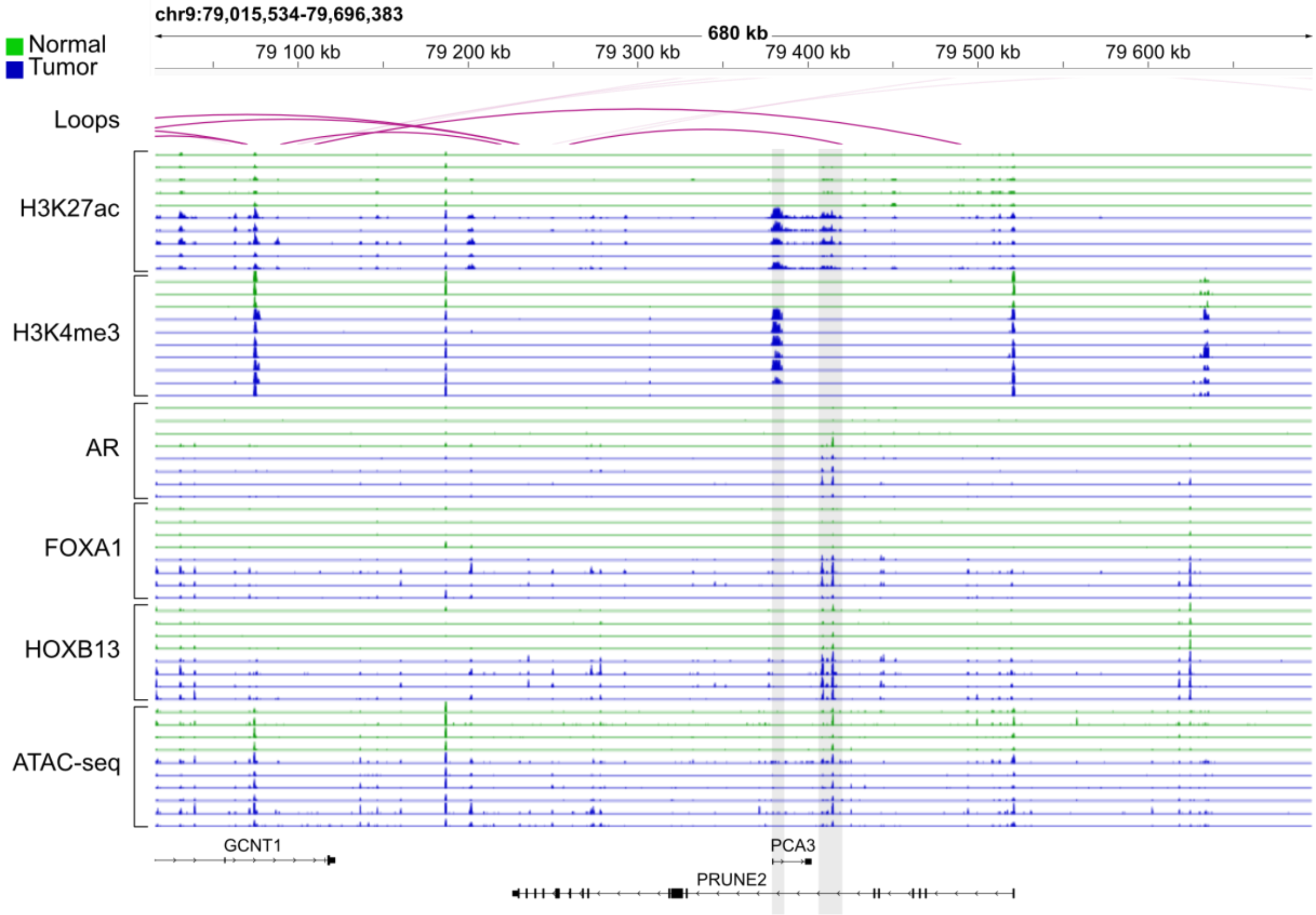


**Figure S16. Epigenetic activation of PCA3 in prostate cancer, related to Figure 4**.

Genomic tracks display ChIP-seq data for histone modifications and transcription factor binding from a public dataset of clinical benign and malignant prostate samples [8]. The PCA3 promoter (left highlight) exhibits cancer-specific enrichment of histone H3 lysine 27 acetylation (H3K27ac) and histone H3 lysine 4 trimethylation (H3K4me3). A downstream enhancer (right highlight) shows elevated H3K27ac signal and recruitment of AR, FOXA1, and HOXB13. At the top, chromatin loops identified from Hi-C data (lifted over to hg19) are indicated.

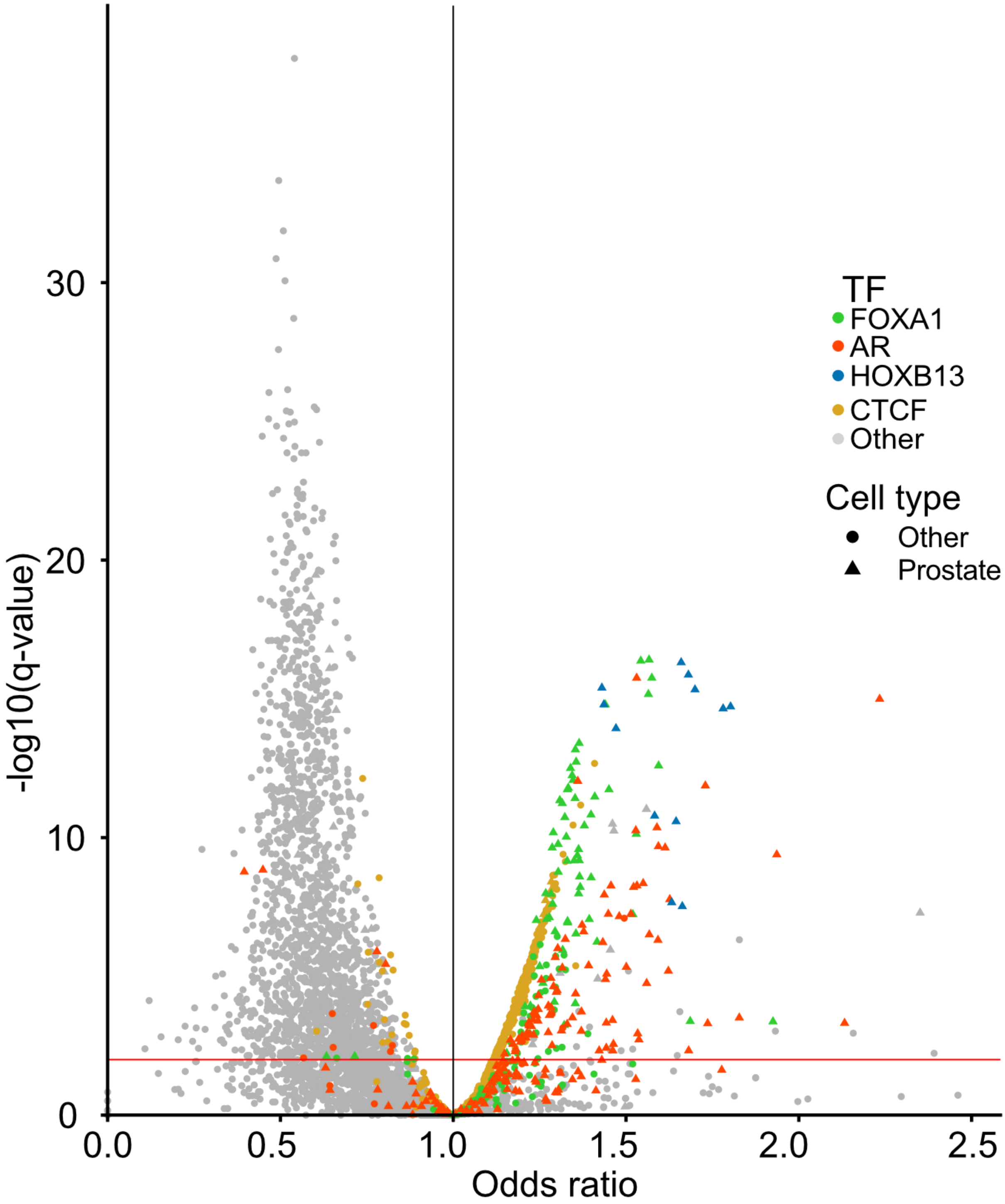


**Figure S17. Enrichment of transcription factor (TF) binding sites in ATAC-peaks located in prostate cancer up-regulated loops, related to Figure 4.**

Each point represents a TF-binding experiment in the unibind database. Enrichment is calculated against a background of ATAC-peaks overlapping the set of unified loops.Triangles represent experiments from prostate cell lines or tissue samples.

A

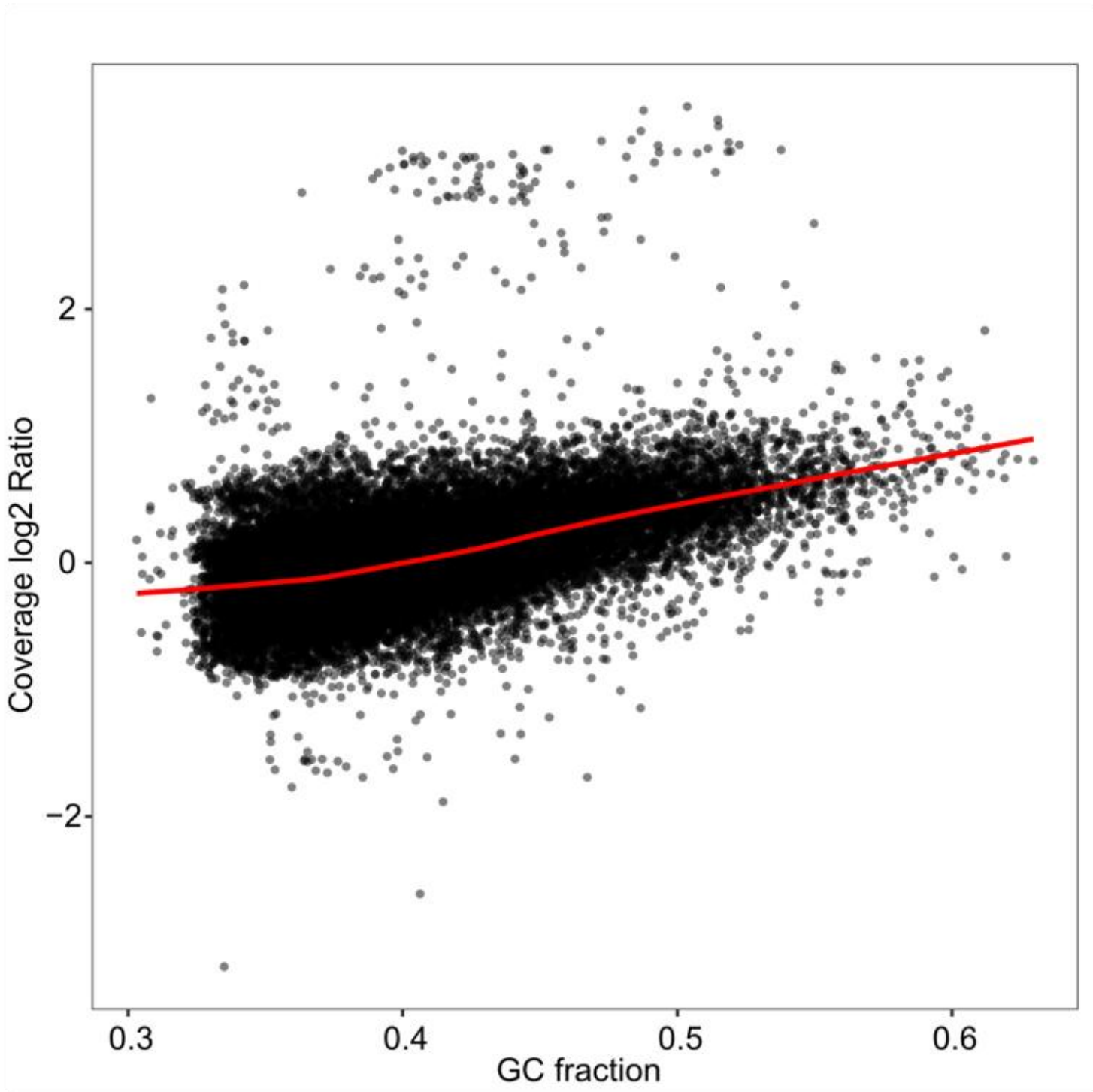


B

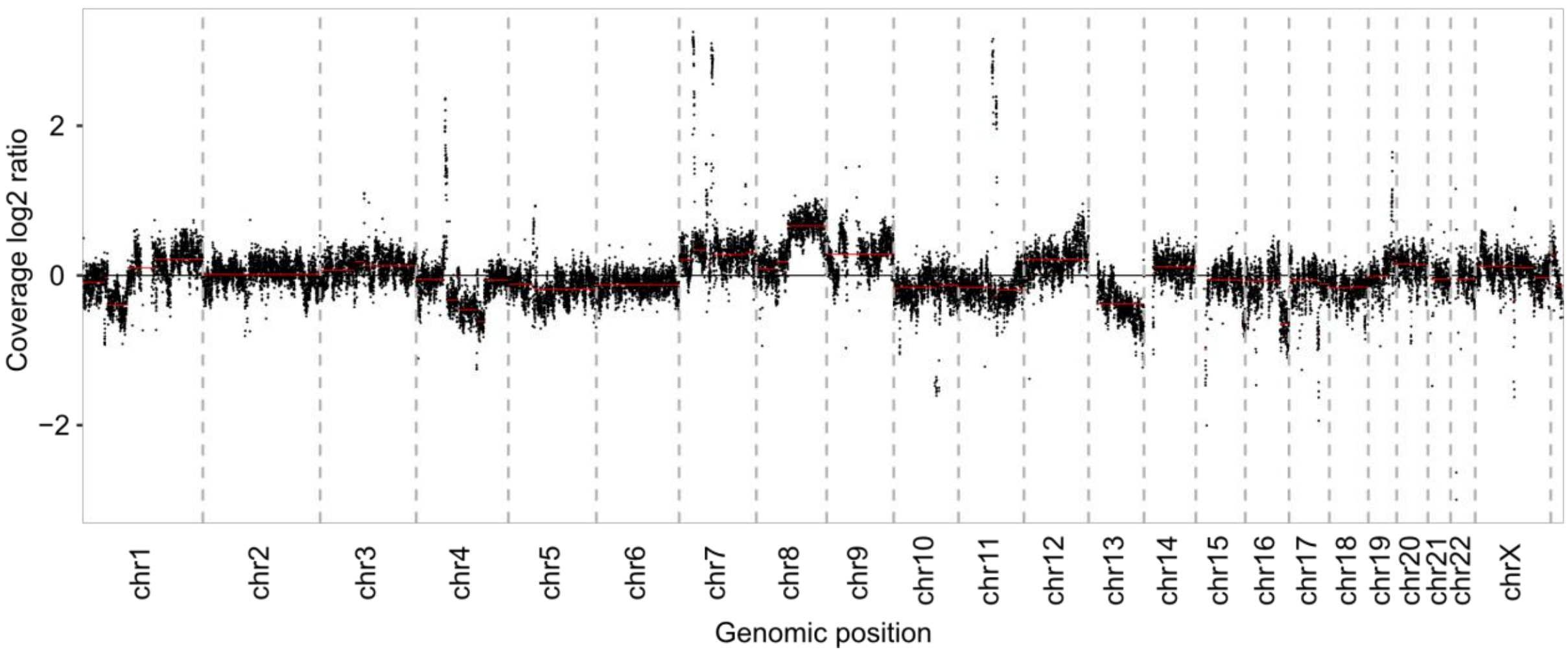


**Figure S18. Copy-number estimation from ATAC-seq data, related to Figure 4.**

The genome was divided into 100 kb bins, and regions within 500 bp of ATAC peaks were masked prior to count assignment. (**a**) Scatter plot of log2 coverage ratios versus GC content illustrating GC-bias correction (see Methods). The estimated GC-dependent bias is shown as a red line. (**b**) Example of a GC-corrected log2 coverage ratio profile from sample CRPC-435.

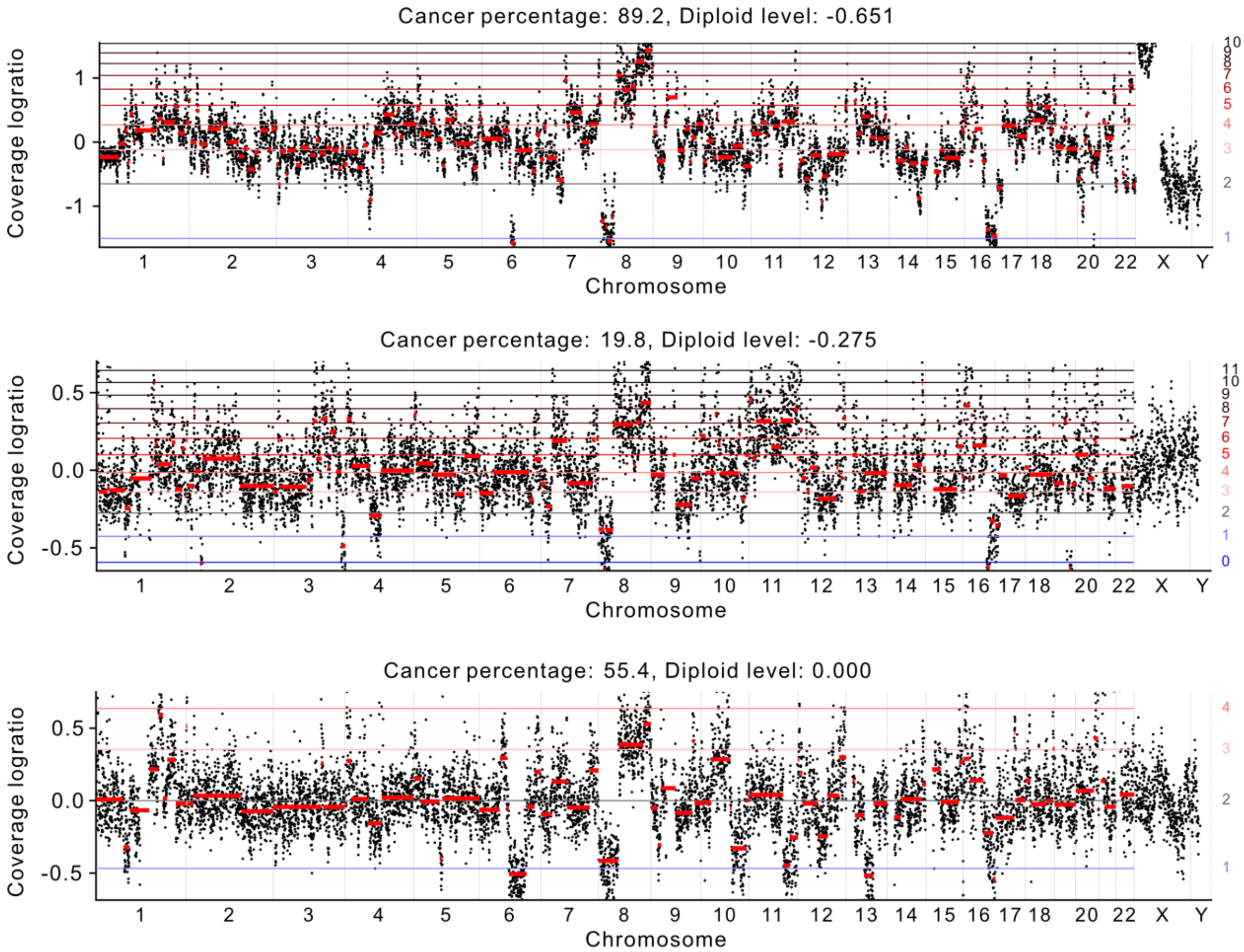


**Figure S19. Tumor fraction estimation from ATAC-seq–derived copy-number profiles, related to Figure 4.**

Three representative examples of estimated tumor fractions and corresponding copy number states are shown. Diploid level is defined as the log2 ratio value associated with the copy-number state of 2.

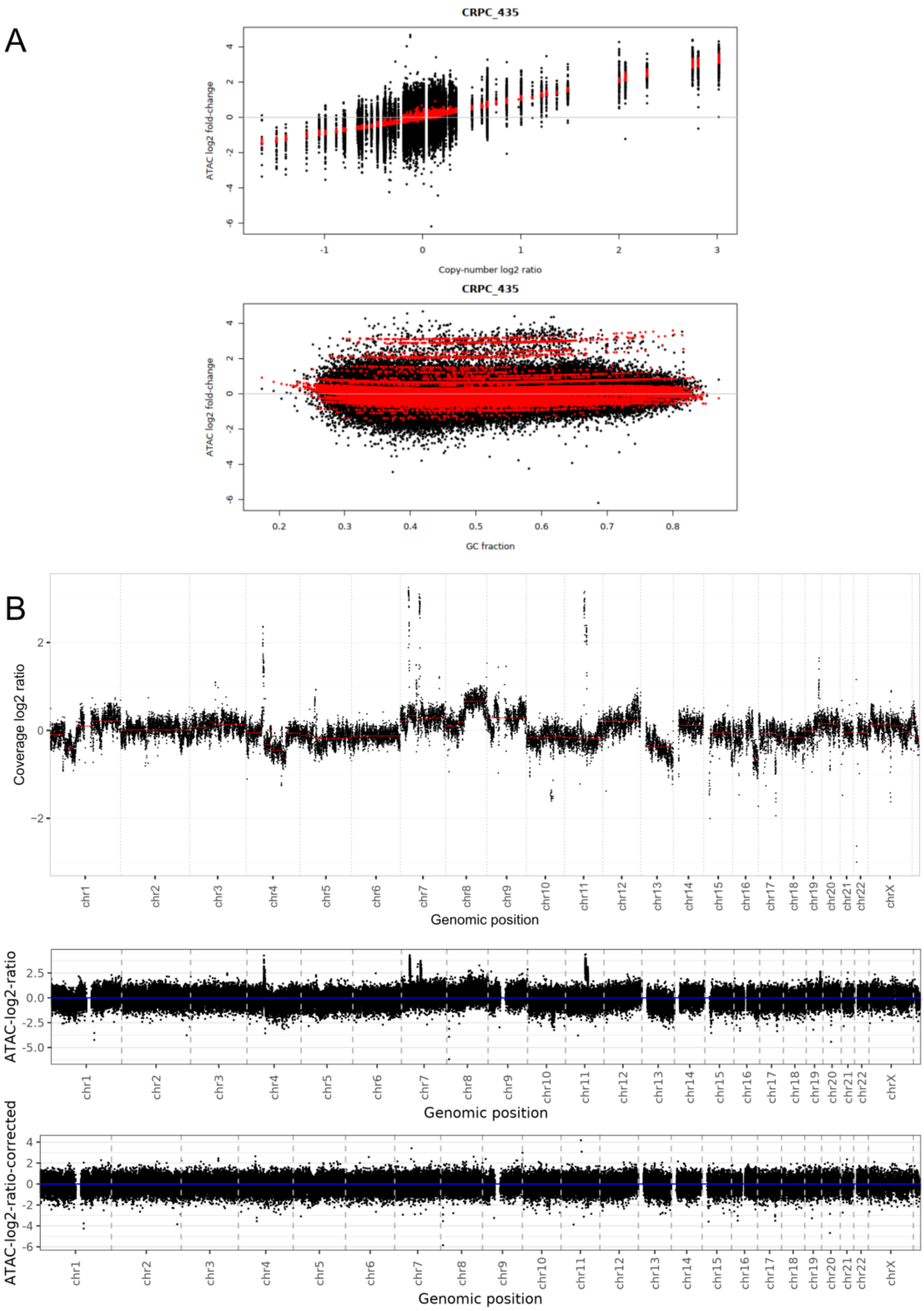


**Figure S20. Copy-number bias correction of ATAC-seq data, related to Figure 4.**

(**a**) Relationship between ATAC-seq log2 fold change (relative to the median value of BPH samples) and copy-number log2 ratio (top) or GC fraction (bottom). Each point represents an ATAC peak, and the estimated bias for each peak is shown in red (see Methods). (b) Example of an ATAC-seq–derived copy-number profile (top) and ATAC log2 fold change before (middle) and after (bottom) copy-number bias correction.

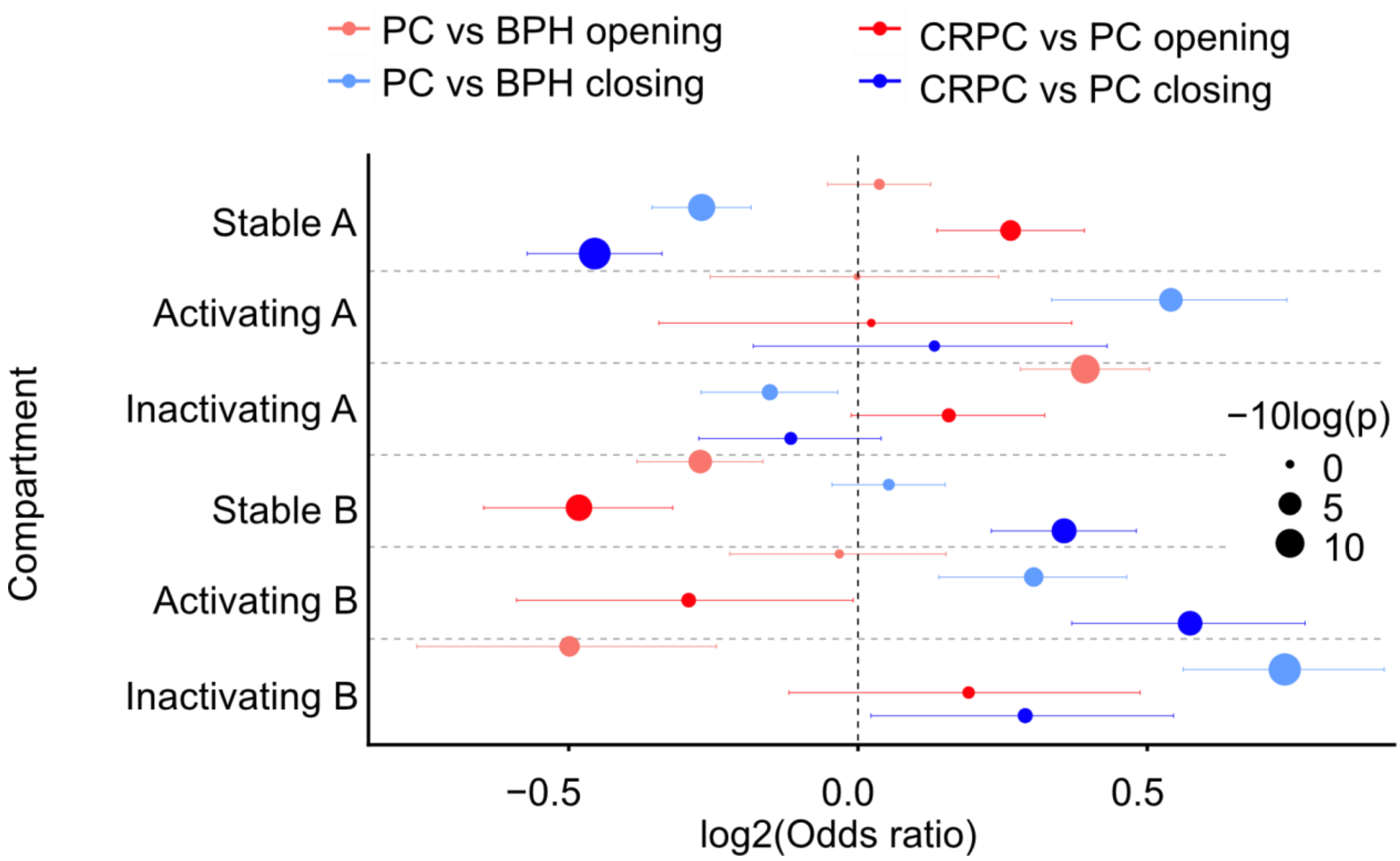


**Figure S21 Association between chromatin accessibility and compartment shifts, related to Figure 4.**

Enrichment of ATAC-seq peaks that open or close in primary prostate cancer (PC) relative to benign prostatic hyperplasia (BPH), and in castration-resistant prostate cancer (CRPC) relative to PC, across genomic compartments. Each compartment is tested against all other genomic regions. Point size represents –log10(*P*-value), and error bars indicate 95% confidence intervals.

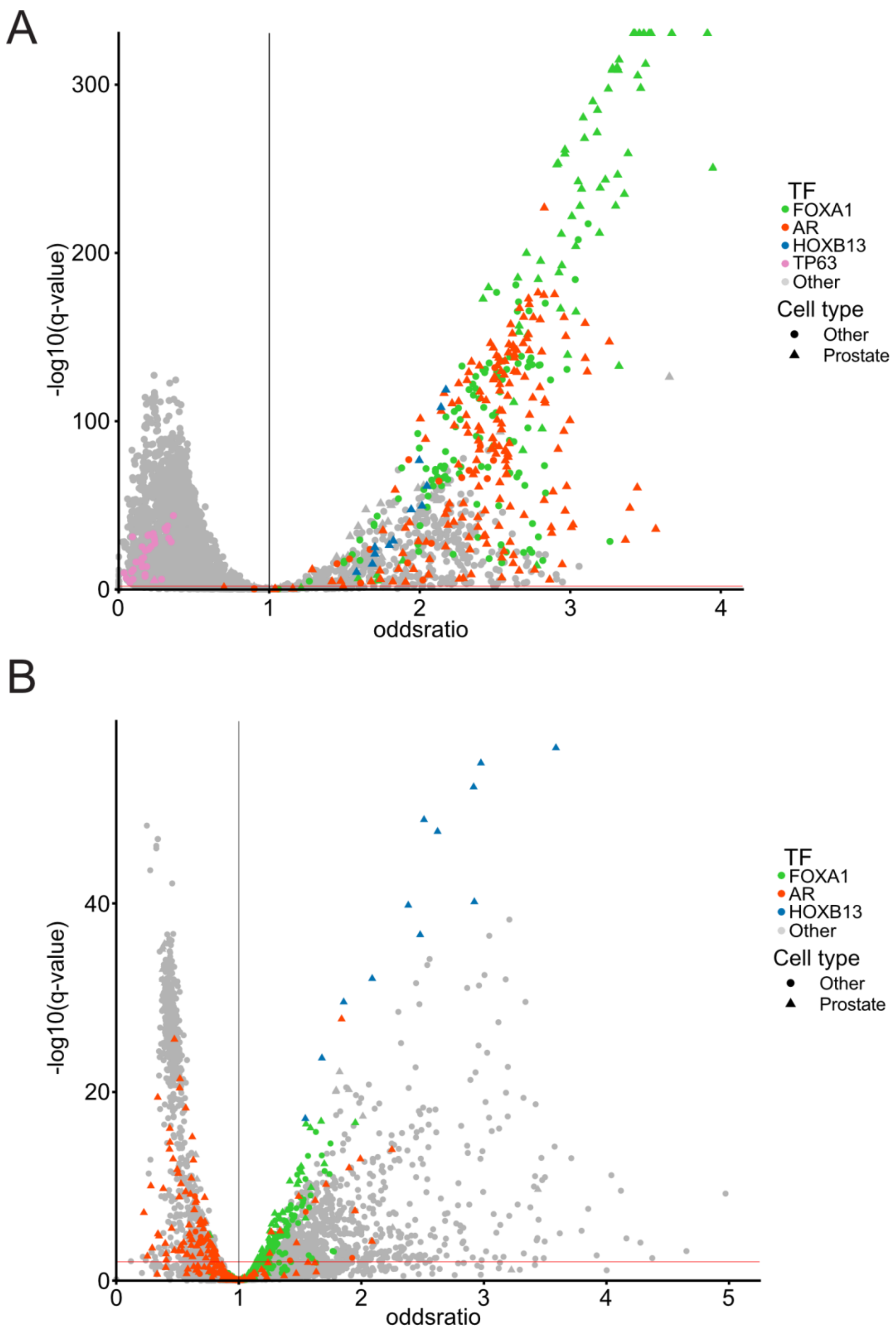


**Figure S22. Transcription factor (TF) binding site enrichment in accessible chromatin, related to Figure 4.**

ATAC-seq peaks upregulated in (**a**) primary prostate cancer relative to benign prostatic hyperplasia and (**b**) castration-resistant prostate cancer relative to primary prostate cancer. Each point represents a TF binding experiment from the UniBind database [9] . Triangles denote experiments performed in prostate-derived cell lines or tissue samples.

# Supplementary references


[1] Quigley, D.A., Dang, H.X., Zhao, S.G., Lloyd, P., Aggarwal, R., Alumkal, J.J., Foye, A., Kothari, V., Perry, M.D., Bailey, A.M., et al. (2018). Genomic hallmarks and structural variation in metastatic prostate cancer. Cell *174*, 758–769.e9.

[2] ICGC/TCGA Pan-Cancer Analysis of Whole Genomes Consortium (2020). Pan-cancer analysis of whole genomes. Nature *578*, 82–93.

[3] Hwang, J.H., Seo, J.-H., Beshiri, M.L., Wankowicz, S., Liu, D., Cheung, A., Li, J., Qiu, X., Hong, A.L., Botta, G., et al. (2019). CREB5 promotes resistance to androgen-receptor antagonists and androgen deprivation in prostate cancer. Cell Rep. *29*, 2355–2370.e6.

[4] Baumgart, S.J., Nevedomskaya, E., Lesche, R., Newman, R., Mumberg, D., and Haendler, B. (2020). Darolutamide antagonizes androgen signaling by blocking enhancer and super-enhancer activation. Mol. Oncol. 14, 2022–2039.

[5] Wang, X., Xu, J., Zhang, B., Hou, Y., Song, F., Lyu, H., and Yue, F. (2021). Genome-wide detection of enhancer-hijacking events from chromatin interaction data in rearranged genomes. Nat. Methods *18*, 661–668.

[6] Kiviaho, A., Eerola, S.K., Kallio, H.M.L., Andersen, M.K., Hoikka, M., Tiihonen, A.M., Salonen, I., Spotbeen, X., Giesen, A., Parker, C.T.A., et al. (2024). Single cell and spatial transcriptomics highlight the interaction of club-like cells with immunosuppressive myeloid cells in prostate cancer. Nat. Commun. *15*, 9949.

[7] Guo, H., Vuille, J.A., Wittner, B.S., Lachtara, E.M., Hou, Y., Lin, M., Zhao, T., Raman, A.T., Russell, H.C., Reeves, B.A., et al. (2023). DNA hypomethylation silences anti-tumor immune genes in early prostate cancer and CTCs. Cell *186*, 2765–2782.e28.

[8] Pomerantz, M.M., Qiu, X., Zhu, Y., Takeda, D.Y., Pan, W., Baca, S.C., Gusev, A., Korthauer, K.D., Severson, T.M., Ha, G., et al. (2020). Prostate cancer reactivates developmental epigenomic programs during metastatic progression. Nat. Genet. *52*, 790–799.

[9] Puig, R.R., Boddie, P., Khan, A., Castro-Mondragon, J.A., and Mathelier, A. (2021). UniBind: maps of high-confidence direct TF-DNA interactions across nine species. BMC Genomics *22*, 482.
